# Supplementary material for: Structure and functional mapping of the KRAB‐KAP1 repressor complex
Source: EMBO J. 2022 Nov 7;41(24):e111179. doi: 10.15252/embj.2022111179 (PMC9753469; doi:10.15252/embj.2022111179)
Supplement: Supplementary file 1 — Expanded View Figures PDF [file EMBJ-41-e111179-s004.pdf]

Expanded View Figures

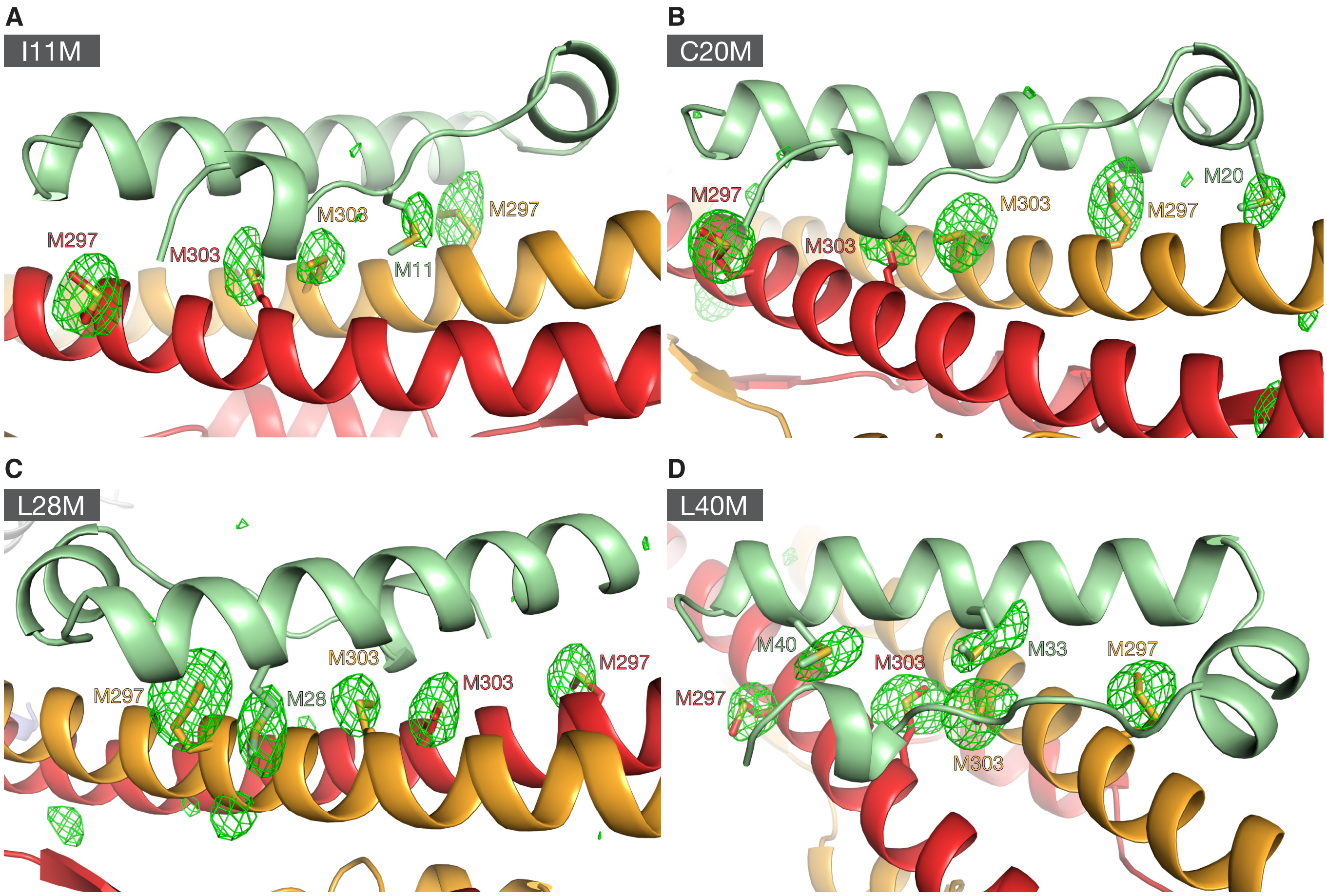

Figure EV1. Anomalous Fourier maps of selenomethionine derivatives of KAP1 RBCC in complex with four different ZNF93 methionine-insertion mutants, related to Fig 1.

A–D X-ray diffraction data were collected at the selenium K absorption edge. Anomalous Fourier maps were contoured at 3.5 σ. The Fourier maps show the positions of selenium atoms of the selenium-substituted methionine residues the KRAB and KAP1 RBCC domains. KAP1 RBCC in complex with ZNF93 KRAB I11M, (A), ZNF93 KRAB C20M, (B), ZNF93 KRAB L28M, (C), ZNF93 KRAB L40M, (D).

© 2022 The Authors

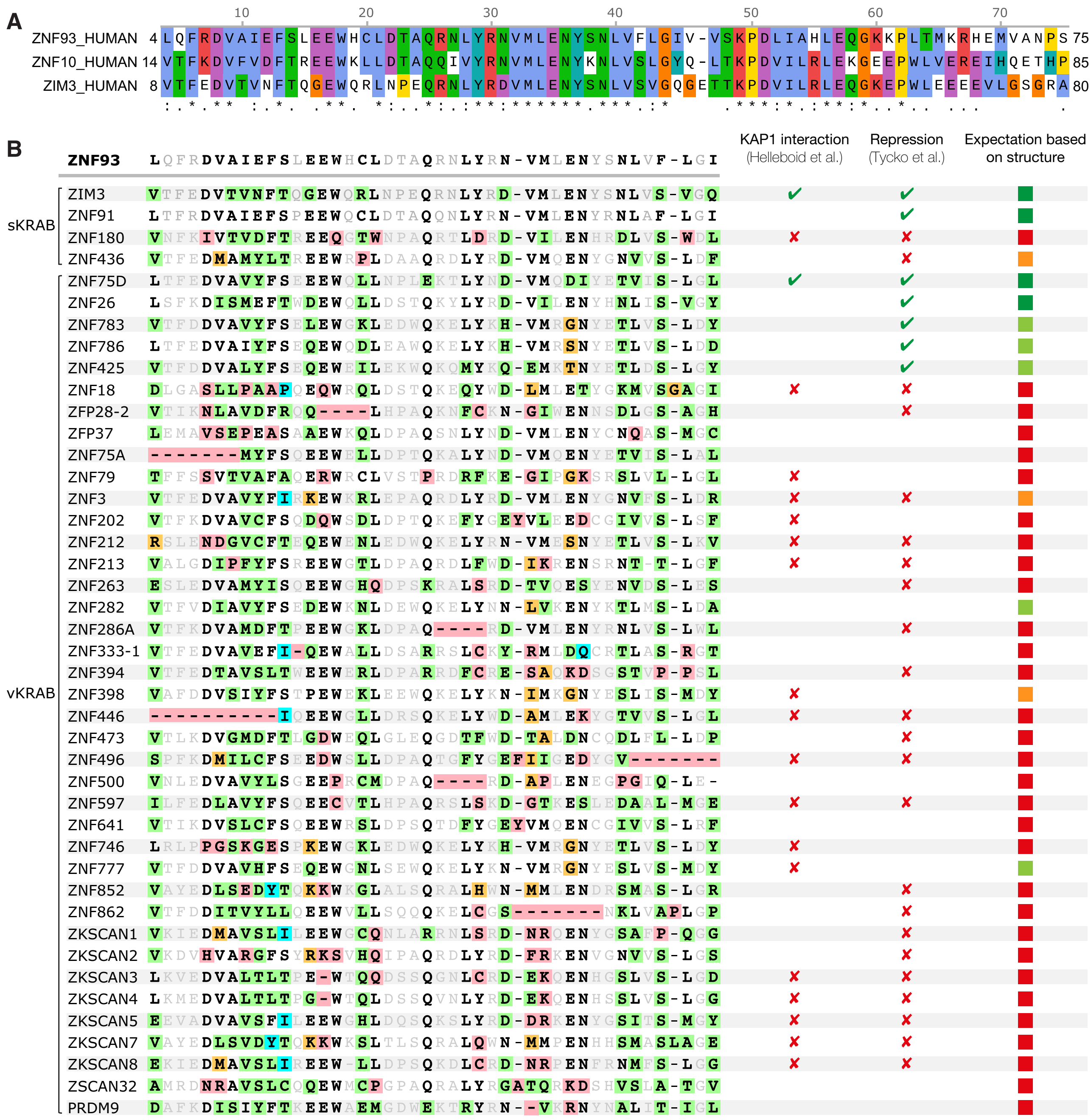

Figure EV2. Amino acid sequence alignments of KRAB domains, related to Fig 3.

A The KRAB domains from ZNF93, ZNF10, and ZIM3. Fused to catalytically inactive Cas9, the KRAB domains of ZNF10 and ZIM3 are used for potent gene repression that can be programmed in a sequence-specific manner via the CRISPRi approach (Gilbert *et al*, 2014; Thakore *et al*, 2015; Alerasool *et al*, 2020).

B Alignment of unusual KRAB domains. Variant KRAB domains (vKRAB, as classified by (Helleboid *et al*, 2019), and two standard KRAB domains (sKRAB) which were non-repressive in a high-throughput screen (Tycko *et al*, 2020), are aligned to the KRAB domain of ZNF93. ZNF93 residues directly contacting KAP1 in the crystal structure are in bold; residues not contacting KAP1 are in gray. KAP1-contacting residues that deviate from the ZNF93 sequence are highlighted in green, orange, or red, depending on whether the mutation is expected to be tolerated, moderately deleterious or highly deleterious, respectively. The right-hand column shows the expectation of whether each KRAB domain will interact with KAP1, based on our structural analysis (green, likely to bind tightly; orange, may still bind; red, unlikely to bind). The repressive sKRAB domains of ZNF91 and ZIM3 are shown for reference.

 © 2022 The Authors

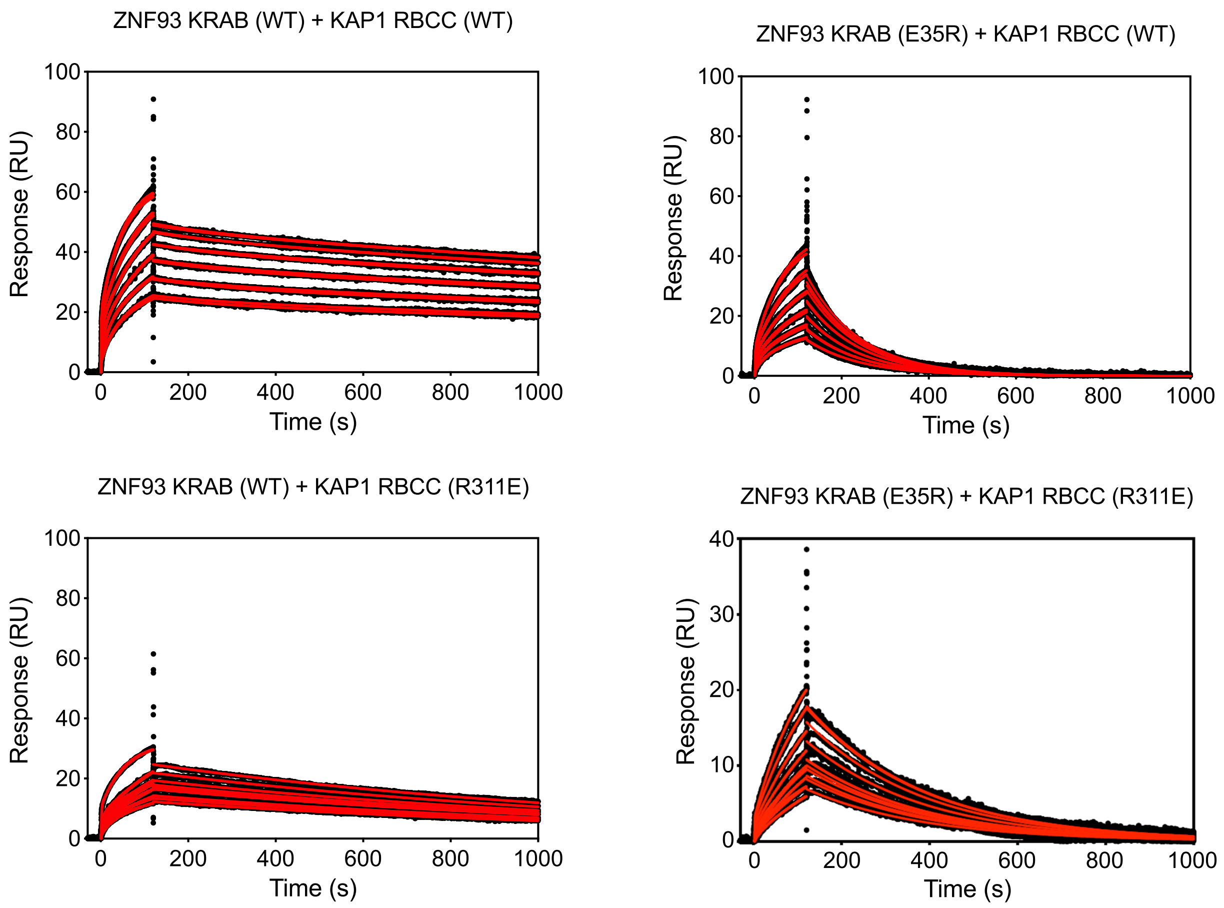

Analyte	Ligand	Phase	k_{off} (s^{-1})	k_{on} ($M^{-1} s^{-1}$)	K_d (nM)
KAP1 RBCC (WT)	ZNF93 KRAB (WT)	major	2.32×10^{-4}	3.11×10^{4}	7.5
		minor	6.1×10^{-3}	3.2×10^{2}	19×10^{3}
KAP RBCC (R311E)	ZNF93 KRAB (WT)	major	8.3×10^{-4}	2.9×10^{4}	29
		minor	-	4.2×10^{2}	2×10^{3}
KAP1 RBCC (WT)	ZNF93 KRAB (E35R)	major	8.0×10^{-3}	4.1×10^{4}	198
		minor	-	1.7×10^{2}	47×10^{3}
KAP1 RBCC (R311E)	ZNF93 KRAB (E35R)	major	3.7×10^{-3}	5.2×10^{4}	71.8
		minor	-	1.05×10^{2}	35.6×10^{3}

Figure EV3. Surface plasmon resonance (SPR) KAP1-KRAB binding assay titration curves, related to Fig 4.

Upper panel: SPR sensorgrams for KAP1 binding to immobilized MBP KRAB. The fits for the association and dissociation kinetics are shown in red. Lower panel: Data were fitted using a biphasic kinetic model with PRISM 9 (GraphPad) to determine rate constants (k_{on}, k_{off}) and binding affinities (K_d).

© 2022 The Authors

Figure EV4. Expression levels and genomic distribution of KAP1 variants in HEK239T cells, related to Fig 5.

A Heatmaps and summary plots illustrating KAP1 ChIP-seq enrichment over H3K9me3 peaks (left) and protein-coding gene promoters (right) genome wide, in KAP1 KO cells and complemented cell lines expressing WT KAP1 or KRAB binding-deficient KAP1 variant K296S/M297S/L300S/V293S (CCmut). TSS, transcriptional start site. The summary plots illustrate mean coverage values (RPKM) for each sample minus the signal of the pooled input sample. Only reads mapping uniquely (MAPQ > 10) were retained. ChIP-seq experiments were run in duplicate.

B Example genome browser snapshots of KAP1 enrichment in KAP1 KO cells complemented cell lines expressing WT or CCmut KAP1. Top, a gene with KRAB-dependent KAP1 binding at the 3′ end and KRAB-independent KAP1 binding at the promoter. Bottom, an LTR transposon bound by KAP1 in a KRAB-dependent manner. The KAP1 KO cells and a pooled input track are shown as controls. Only reads mapping uniquely (MAPQ > 10) were retained. Scales are in RPKM, reads per kilobase per million.

C Western blot of WT, KAP1 KO, and KAP1-complemented KAP1 KO HEK293T cells. These cells were used for CUT&RUN genomic profiling (Figs 5 and EV5).

D Confocal immunofluorescence microscopy of the HEK293T cells used for CUT&RUN genomic profiling stained with anti-KAP1 antibody and DAPI nuclear stain. Scale bars, 10 μm.

E Co-immunoprecipitation of KAP1 and SETDB1. KAP1 was immunoprecipitated from HEK293T KAP1 KO cells stably expressing 3xFLAG-KAP1 (WT or CCmut) using ANTI-FLAG M2 beads. The experiment was performed either in the presence or absence of the SUMO protease inhibitor N-ethylmaleimide (NEM). Uncropped blots available in Source Data.

Source data are available online for this figure.

© 2022 The Authors

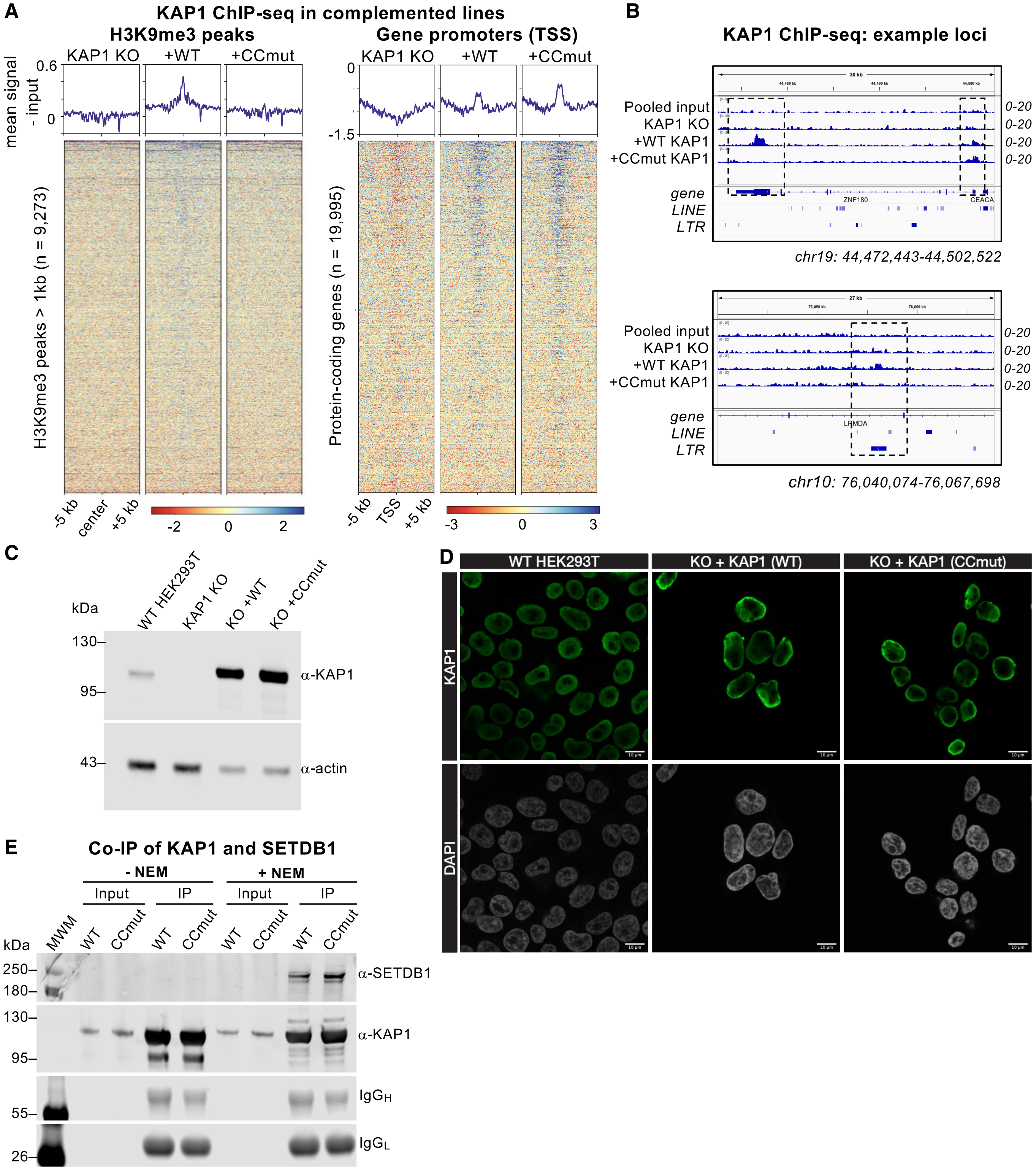
A
KAP1 ChIP-seq in complemented lines
H3K9me3 peaks
Gene promoters (TSS)
KAP1 KO
+WT
+CCmut
mean signal - input
0.6
0
-1.5
H3K9me3 peaks > 1kb (n = 9,273)
Protein-coding genes (n = 19,995)
-5 kb
center
+5 kb
TSS
-2
2
-3
3
B
KAP1 ChIP-seq: example loci
Pooled input
KAP1 KO
+WT KAP1
+CCmut KAP1
gene
LINE
LTR
0-20
ZNF180
CEACA
chr19: 44,472,443-44,502,522
LRMDA
chr10: 76,040,074-76,067,698
C
kDa
WT HEK293T
KAP1 KO
KO +WT
KO +CCmut
130–
95–
43–
α-KAP1
α-actin
D
WT HEK293T
KO + KAP1 (WT)
KO + KAP1 (CCmut)
KAP1
DAPI
E
Co-IP of KAP1 and SETDB1
- NEM
+ NEM
Input
IP
MWM
WT
CCmut
kDa
250–
180–
130–
95–
55–
26–
α-SETDB1
α-KAP1
IgGH
IgGL

Figure EV4.

Figure EV5. Genome-wide analysis of H3K9me3 distribution in cells expressing wild-type and KRAB binding-deficient variants of KAP1, Related to Fig 5.

A Pairwise quantifications of H3K9me3 CUT&RUN counts for KAP1-complemented cells ($n = 2$) versus KAP1 KO cells ($n = 2$) over reference H3K9me3 peaks called in the parental control cell line. Only reads mapping uniquely (MAPQ > 10) were retained. Gray indicates significant enrichment of signal (cut-off: $P < 0.0001$; fold-change > 3) in the complemented line.

B Representative H3K9me3 distribution (over the hg38 reference) in the presence of different KAP1 variants at three different types of KAP1-independent loci: an intronic LINE-1 (L1PA) element bound by the HUSH complex (upper); a HUSH-bound long exon (middle); and a centromeric region (lower). A control IgG track from parent HEK293T cells is shown for comparison. WT, wild-type; CCmut, KRAB binding-deficient KAP1 variant. Only reads mapping uniquely (MAPQ > 10) were retained. Scales are in RPKM, reads per kilobase per million. Experiments were run in duplicate with similar results.

C Heatmaps and summary plots illustrating H3K9me3 levels over H3K9me3 peaks at HUSH-bound loci (Douse *et al*, 2020; Seczynska *et al*, 2022), in cells expressing different KAP1 variants. Peaks were called on control cells using SEACR in stringent mode (Meers *et al*, 2019). The summary plots illustrate mean values for each sample. Only reads mapping uniquely (MAPQ > 10) were retained.

D Quantitative comparison of H3K9me3 changes upon KAP1 knockout over loci regulated by HUSH (Douse *et al*, 2020; Seczynska *et al*, 2022) versus those regulated by KAP1 (as defined in this study). The central bands denote the medians. Boxes represent the interquartile range (IQR). Whiskers extend 1.5x IQR beyond the box. Statistical test: Wilcoxon rank sum test with continuity correction.

E Bar plot illustrating the distribution of loci where H3K9me3 was not reduced in KAP1 KO cells compared with the parental control cell line (KAP1-independent loci).

 © 2022 The Authors

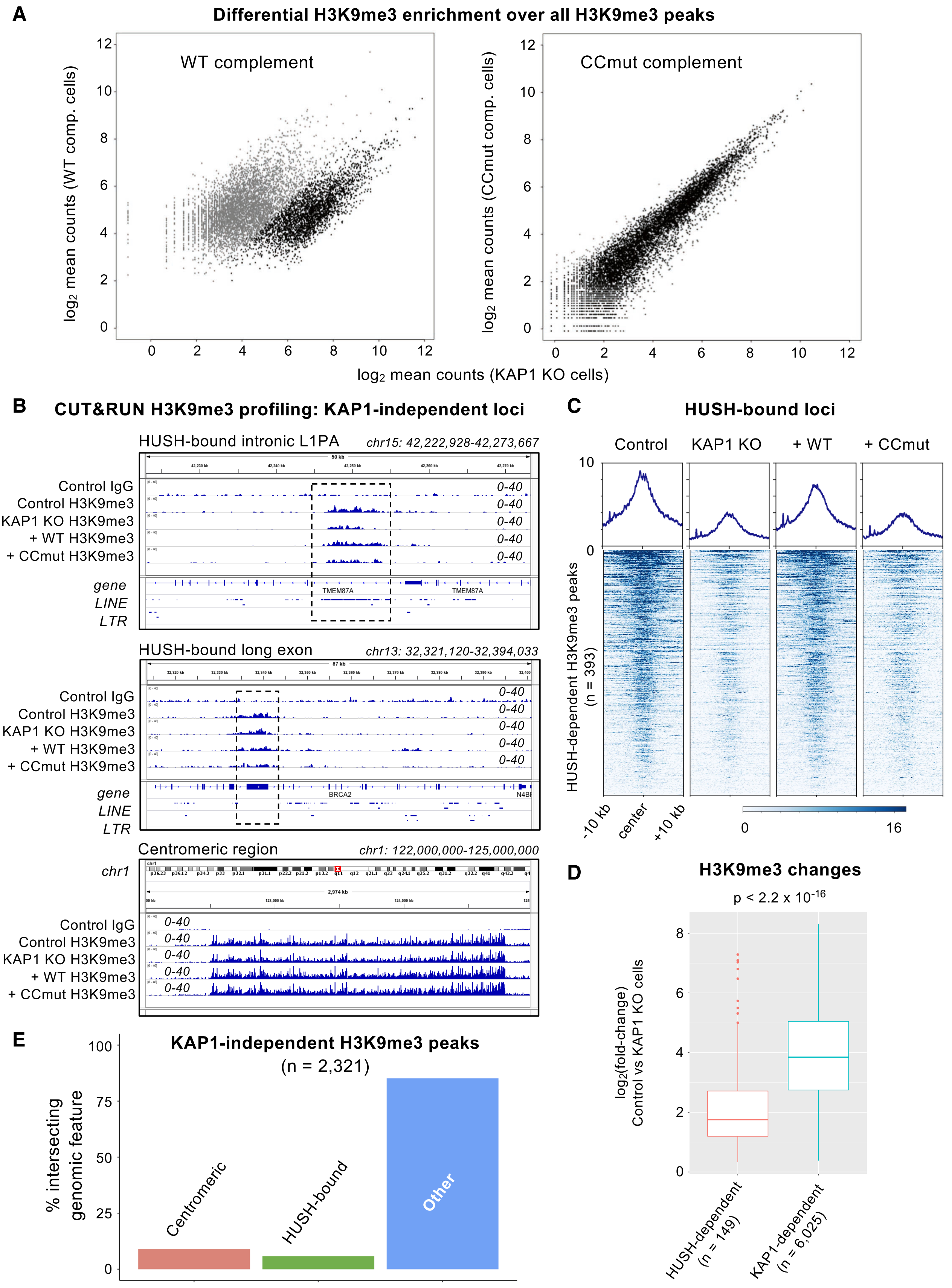
A
Differential H3K9me3 enrichment over all H3K9me3 peaks
WT complement
CCmut complement
log2 mean counts (WT comp. cells)
log2 mean counts (CCmut comp. cells)
log2 mean counts (KAP1 KO cells)
B
CUT&RUN H3K9me3 profiling: KAP1-independent loci
HUSH-bound intronic L1PA
chr15: 42,222,928-42,273,667
Control IgG
Control H3K9me3
KAP1 KO H3K9me3
+ WT H3K9me3
+ CCmut H3K9me3
gene
LINE
LTR
TMEM87A
HUSH-bound long exon
chr13: 32,321,120-32,394,033
BRCA2
Centromeric region
chr1: 122,000,000-125,000,000
chr1
C
HUSH-bound loci
Control
KAP1 KO
+ WT
+ CCmut
HUSH-dependent H3K9me3 peaks (n = 393)
-10 kb
center
+10 kb
D
H3K9me3 changes
p < 2.2 x 10-16
log2(fold-change) Control vs KAP1 KO cells
HUSH-dependent (n = 149)
KAP1-dependent (n = 6,025)
E
KAP1-independent H3K9me3 peaks
(n = 2,321)
% intersecting genomic feature
Centromeric
HUSH-bound
Other

Figure EV5.
