# Supplementary figures and images for: Structure and functional mapping of the KRAB‐KAP1 repressor complex
Source: EMBO J. 2022 Nov 7;41(24):e111179. doi: 10.15252/embj.2022111179 (PMC9753469; doi:10.15252/embj.2022111179)

Fig EV4C

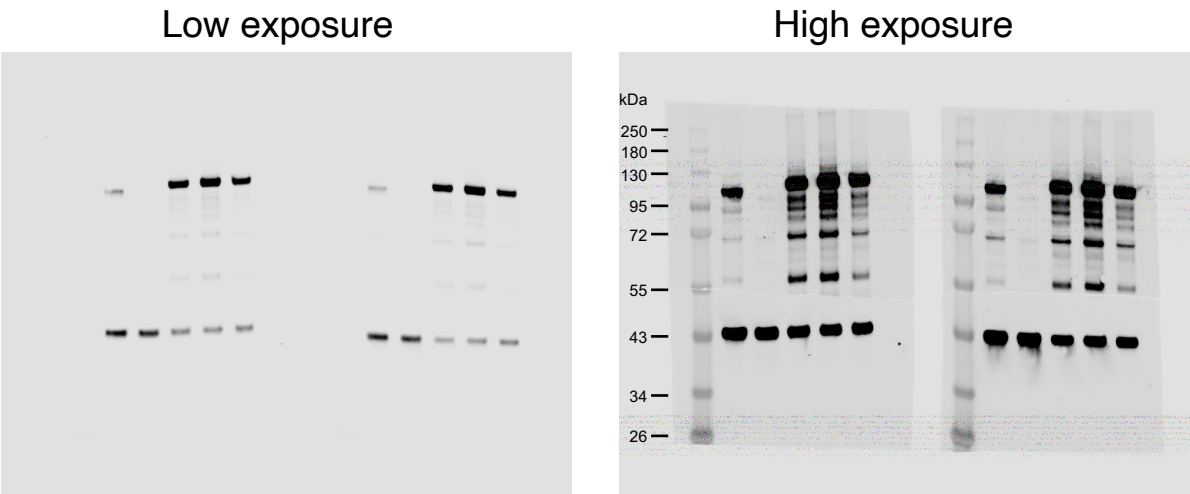

Fig EV4E

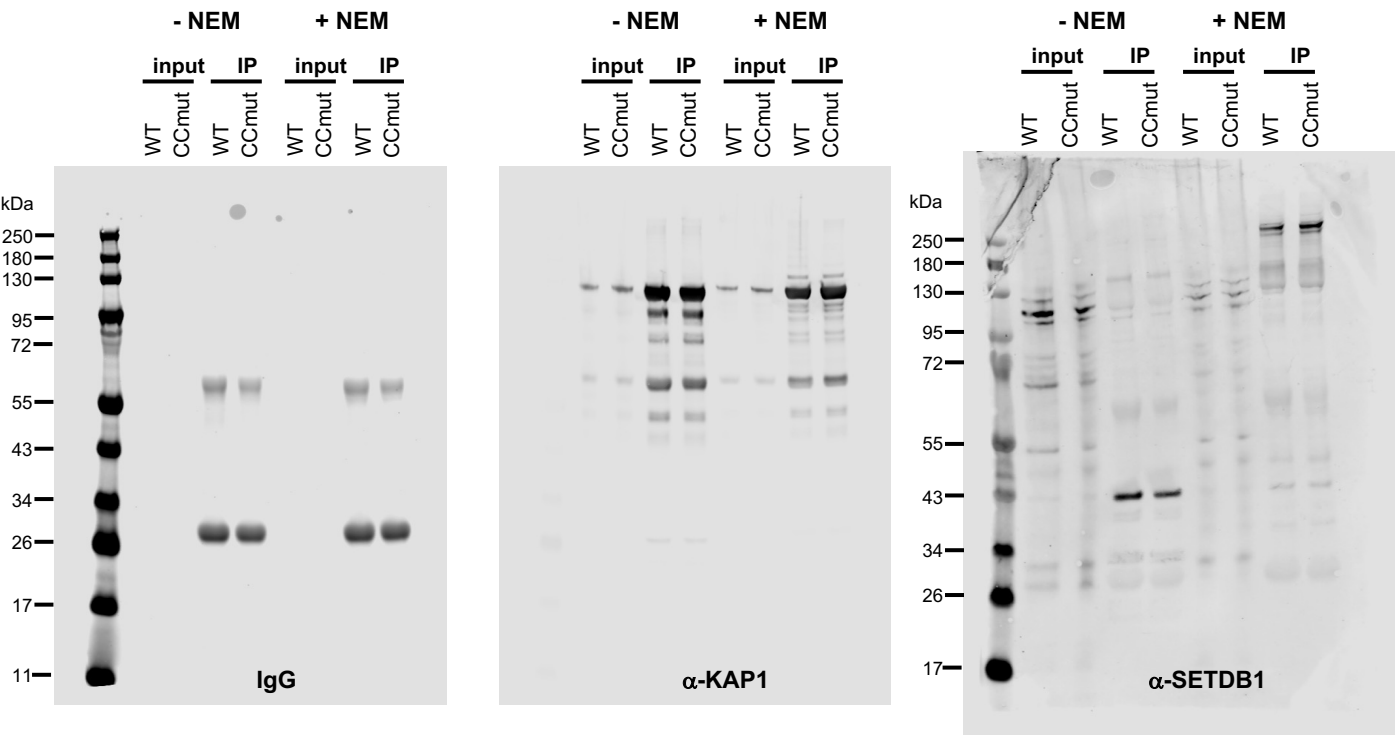

Supplement: Supplementary file 2 — Source Data for Expanded View [file EMBJ-41-e111179-s002.zip › SD_Fig_EV4/source_data_EV4.pdf]

**Fig 4C**

Low exposure

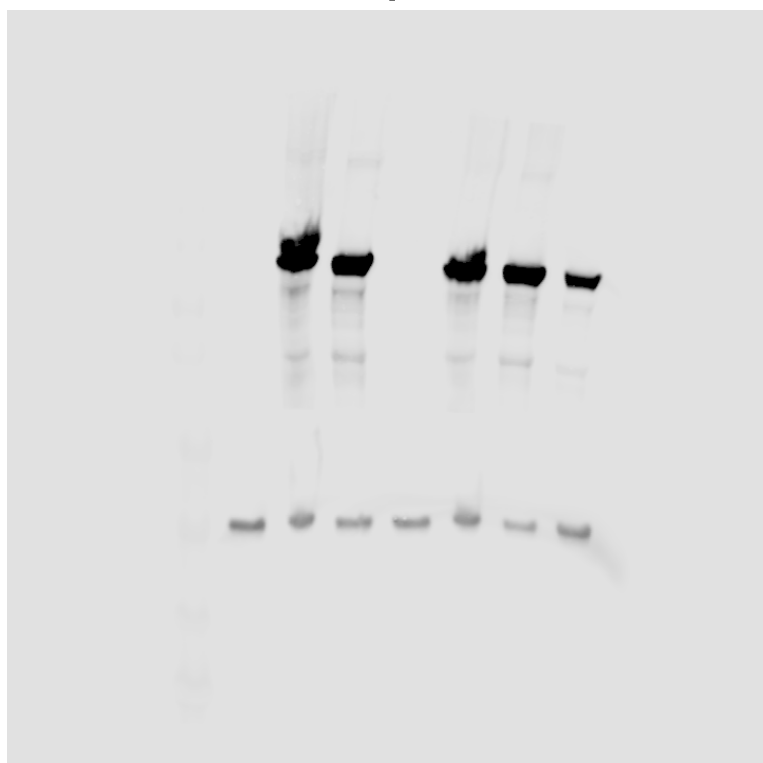

High exposure

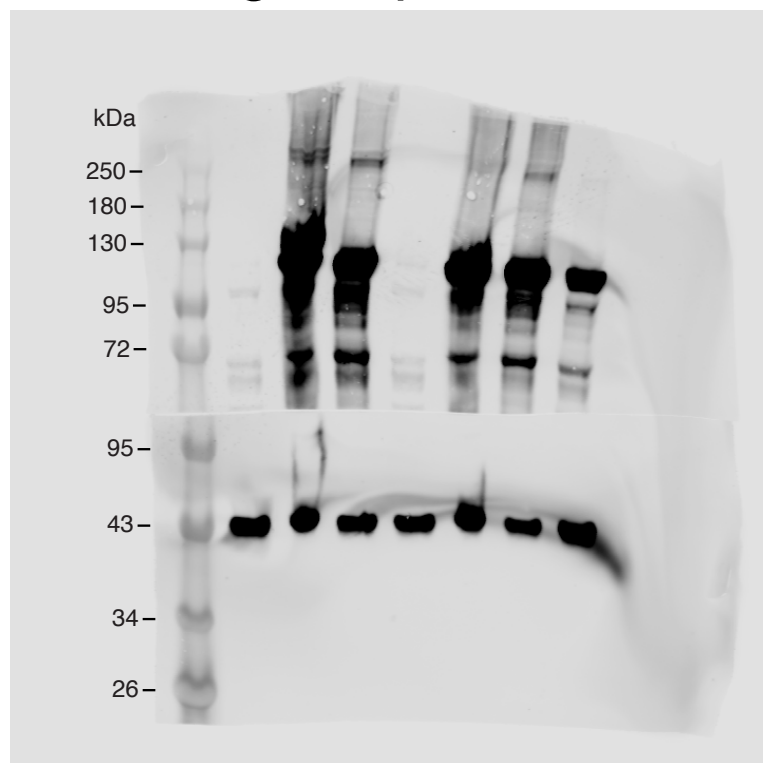

**Fig 4D**

Low exposure

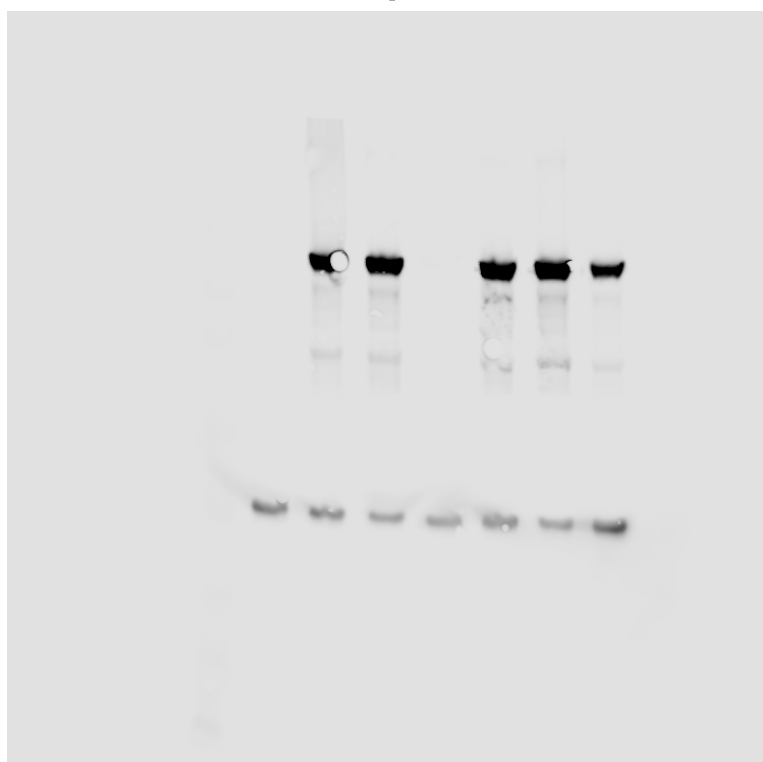

High exposure

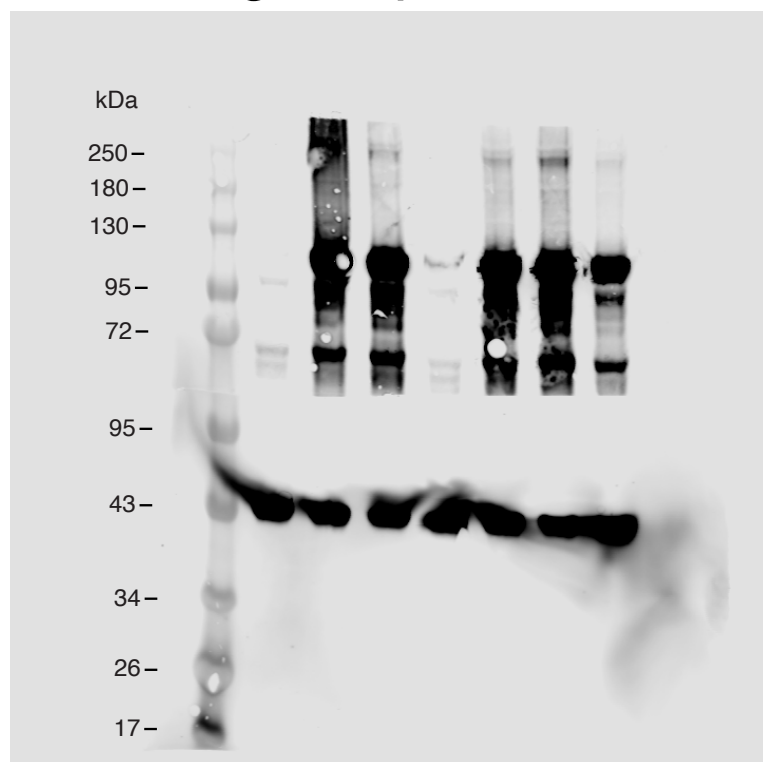

Supplement: Supplementary file 4 — Source Data for Figure 4 [file EMBJ-41-e111179-s003.zip › SD_Fig_4/source_data_fig4.pdf]
