# Supplementary material for: Structure and functional mapping of the KRAB‐KAP1 repressor complex
Source: EMBO J. 2022 Nov 7;41(24):e111179. doi: 10.15252/embj.2022111179 (PMC9753469; doi:10.15252/embj.2022111179)
Supplement: Supplementary file 3 — PDF+ [file EMBJ-41-e111179-s005.pdf]

Article

SOURCE DATA | TRANSPARENT PROCESS | OPEN ACCESS

THE EMBO JOURNAL

Structure and functional mapping of the KRAB-KAP1 repressor complex

Guido A Stoll[1,2], Ninoslav Pandiloski[3], Christopher H Douse[3] & Yorgo Modis[1,2,*]

Abstract

Transposable elements are a genetic reservoir from which new genes and regulatory elements can emerge. However, expression of transposable elements can be pathogenic and is therefore tightly controlled. KRAB domain-containing zinc finger proteins (KRAB-ZFPs) recruit the co-repressor KRAB-associated protein 1 (KAP1/TRIM28) to regulate many transposable elements, but how KRAB-ZFPs and KAP1 interact remains unclear. Here, we report the crystal structure of the KAP1 tripartite motif (TRIM) in complex with the KRAB domain from a human KRAB-ZFP, ZNF93. Structure-guided mutations in the KAP1-KRAB binding interface abolished repressive activity in an epigenetic transcriptional silencing assay. Deposition of H3K9me3 over thousands of loci is lost genome-wide in cells expressing a KAP1 variant with mutations that abolish KRAB binding. Our work identifies and functionally validates the KRAB-KAP1 molecular interface, which is critical for a central transcriptional control axis in vertebrates. In addition, the structure-based prediction of KAP1 recruitment efficiency will enable optimization of KRABs used in CRISPRi.

Keywords CRISPRi; H3K9me3; heterochromatin; Krüppel-associated box; Transposable element
Subject Categories Chromatin, Transcription & Genomics; Structural Biology
DOI 10.15252/embj.2022111179 | Received 15 March 2022 | Revised 18 October 2022 | Accepted 20 October 2022
The EMBO Journal (2022) e111179

Introduction

More than half of the human genome consists of transposable elements (TEs; Friedli & Trono, 2015). TEs can be acquired when viral DNA integrates into the genome of a host germline cell. These endogenous viral elements (EVEs) can retain the ability to replicate by expressing the viral reverse transcriptase and integrase, which convert EVE transcripts into DNA and integrate the DNA into the host genome (Friedli & Trono, 2015). Other TEs, such as LINEs (long interspersed nuclear elements), replicate via a similar retrotransposition mechanism, but the distinct sequence and biochemical activities of LINE proteins suggest they evolved from early eukaryotic, rather than viral genetic elements (Goodier, 2016). Approximately 100 LINEs are replication competent and 2–5% of newborn children have a new LINE insertion (Goodier, 2016). Although most human EVEs have lost transposition activity, some of the most recently acquired human endogenous retrovirus (HERVs) retain the potential to be transcribed, translated, and transposed (Grow *et al*, 2015; Li *et al*, 2015).

Transcription, translation, and transposition of TEs are potentially pathogenic, particularly in embryogenesis, chronic infection, and stress responses, when pro-transcriptional chromatin modifications are enriched (Azebi *et al*, 2019). Accumulation of TE-derived nucleic acids is associated with autoimmune diseases including geographic atrophy, lupus and Sjögren's syndrome (Hung *et al*, 2015; Goodier, 2016). Aberrant expression of HERV proteins is associated with cancer and neurodegeneration (Li *et al*, 2015; Kremer *et al*, 2019). When transposition events disrupt tumor suppressor genes or enhance oncogene expression they contribute to cancer (Lamprecht *et al*, 2010; Hancks & Kazazian, 2016). Gene disruption by TE transposition is also linked to genetic disorders such as hemophilia and cystic fibrosis (Hancks & Kazazian, 2016). The activities of TEs must therefore be tightly regulated.

Counterbalancing these risks, TEs are a genetic reservoir from which new genes and regulatory elements can emerge. TEs drive the evolution of transcriptional networks by spreading transcription factor binding sites, promoters, and other regulatory elements (Friedli & Trono, 2015; Chuong *et al*, 2016). Pluripotency-associated transcription factors required for cell fate determination bind to sites within TEs (Friedli & Trono, 2015). Some TE genes have also been coopted to fulfill important cellular functions. For example, TE-derived proteins catalyze V(D)J recombination (Zhou *et al*, 2004), trophoblast fusion in placental development (Dupressoir *et al*, 2012; Friedli & Trono, 2015), and cell-to-cell mRNA transfer required for synaptic plasticity and memory formation (Pastuzyn *et al*, 2018).

The primary mechanism cells have evolved to control TEs is epigenetic transcriptional silencing. In tetrapod vertebrates, Krüppel-associated box zinc-finger proteins (KRAB-ZFPs) and KRAB-associated protein 1 (KAP1, also known as TRIM28 or TIF1β) are key repressors of TEs (Rowe *et al*, 2010; Helleboid *et al*, 2019). KRAB-ZFPs are the largest mammalian transcription factor family. Humans have 350–400 KRAB-ZFPs, the majority of which recognize

1 Molecular Immunity Unit, Department of Medicine, MRC Laboratory of Molecular Biology, University of Cambridge, Cambridge, UK
2 Cambridge Institute of Therapeutic Immunology & Infectious Disease (CITIID), University of Cambridge School of Clinical Medicine, Cambridge, UK
3 Department of Experimental Medical Science, Lund Stem Cell Center, Lund University, Lund, Sweden
*Corresponding author. Tel: +44 1223 267282; E-mail: ymodis@mrc-lmb.cam.ac.uk

© 2022 The Authors. Published under the terms of the CC BY 4.0 license.

specific TE-derived DNA sequences with their tandem variable zinc-finger arrays (Jacobs *et al*, 2014; Imbeault *et al*, 2017). The expansion in the number of KRAB-ZFPs in mammals has been attributed to evolutionary pressure from TEs mutating to escape recognition, resulting in an arms race between hosts and TEs (Jacobs *et al*, 2014). Because many sequences targeted by KRAB-ZFPs have been repurposed as promoters or enhancers during the course of evolution, some of the older KRAB-ZFPs now regulate physiologically important processes such as genomic imprinting, embryogenesis, brain development, and immunity (Imbeault *et al*, 2017; Tie *et al*, 2018; Azebi *et al*, 2019; Tycko *et al*, 2020; Li *et al*, 2021; Johansson *et al*, 2022). Once bound to DNA (all but a few), KRAB-ZFPs recruit corepressor KAP1 via the conserved KRAB domain (Friedman *et al*, 1996; Kim *et al*, 1996; Moosmann *et al*, 1996; Helleboid *et al*, 2019; Tycko *et al*, 2020). Disrupting KAP1 function is lethal early in embryonic development (Cammas *et al*, 2000).

KRAB domains typically contain a KRAB-A box (40–50 amino acids) necessary and sufficient for KAP1-dependent repression and a KRAB-B box (20–25 amino acids) with an accessory role (Margolin *et al*, 1994; Witzgall *et al*, 1994; Peng *et al*, 2007; Tycko *et al*, 2020). Solution NMR studies of the KRAB-A box from a mouse KRAB-ZFP generated a partly α-helical, partly disordered structural ensemble (Saito *et al*, 2003). A complete mutational scan of the KRAB domain identified 12 residues in the KRAB-A box where mutations abolished silencing, along with a few residues where substitutions enhanced silencing (Tycko *et al*, 2020). Most of the residues required for silencing were required for KAP1 binding in a recombinant protein binding assay (Peng *et al*, 2009) and predicted to cluster together in a structural model of the KRAB-A box (Tycko *et al*, 2020). KRAB domains with the strongest KAP1 binding and silencing activities are among the most powerful transcriptional repressors. Fused to inactive Cas9 in the CRISPR interference (CRISPRi) approach, KRAB domains allow potent programmable gene repression (Gilbert *et al*, 2014; Thakore *et al*, 2015; Alerasool *et al*, 2020).

At its target DNA loci, KAP1 functions as a recruitment platform for repressive chromatin-modifying enzymes including histone H3K9 methyltransferase SETDB1, heterochromatin protein HP1, and the nucleosome remodeling and deacetylase (NuRD) complex (Schultz *et al*, 2001, 2002). We showed previously that KAP1 dimerizes via the coiled-coil domain in its RING, B-box zinc finger and Coiled-Coil (RBCC) motif (Stoll *et al*, 2019). One KAP1 dimer binds a single KRAB domain. A set of four structure-based mutations in the coiled-coil domain, near the dyad of the KAP1 RBCC dimer, abolished KRAB binding and transcriptional silencing, suggesting these mutations map to the KRAB binding site (Stoll *et al*, 2019). However, the structure of the KRAB-KAP1 interaction remains unknown. Here, we report the crystal structure of the KAP1 RBCC in complex with the KRAB domain from ZNF93, a KRAB-ZFP that represses LINE-1 elements in primates (Jacobs *et al*, 2014). The structure provides a three-dimensional atlas of the KRAB-KAP1 binding interface. We use an epigenetic gene silencing assay to confirm that KAP1 residues forming key contacts with the KRAB domain are essential for silencing. Immunoprecipitation experiments show that SETDB1 recruitment by KAP1 is dependent on SUMOylation but independent of KRAB binding. Epigenomic profiling shows that KAP1 chromatin binding and deposition of repressive H3K9me3 marks is disrupted genome-wide in cells expressing a KAP1 variant with mutations at the KRAB binding interface. Our work identifies and functionally validates the KRAB-KAP1 molecular interface, at the nexus of a transcriptional control axis that is vital to vertebrates and underpins programmable gene repression by CRISPRi.

Results

Crystallographic structure determination of a KRAB-KAP1 core complex

To elucidate the molecular basis of transcriptional regulation by KRAB-ZFPs, we determined the crystal structure of the KRAB domain from human ZNF93, a KRAB-ZFP that represses LINE-1 elements (Jacobs *et al*, 2014), in complex with the RBCC domain of KAP1. Structure determination was technically challenging. Initial crystallization trials with the ZNF93 KRAB–KAP1 RBCC complex were unsuccessful. Fusion of bacteriophage T4 lysozyme to the N-terminus of KAP1 RBCC-induced crystallization, but the crystals diffracted X-rays poorly, precluding structure determination. The crystal quality was improved by deleting the flexible B-box 1 domain of KAP1 and adding the CUE1 domain from the chromatin remodeler SMARCAD1, which binds to the coiled-coil domain of KAP1 (Ding *et al*, 2018; Lim *et al*, 2019), to the complex (Fig 1A). The resulting crystals allowed collection of X-ray diffraction data extending to 2.8 Å resolution (Table 1). The structure was determined by molecular replacement (see Materials and Methods), but the electron density in the KRAB domain, particularly for side chains, was weaker than for the rest of the complex (Fig 1B). The directionality and sequence register of the KRAB backbone remained too ambiguous to allow an atomic model to be built *de novo*. However, guided by the AlphaFold2 model of the ZNF93 KRAB domain alone and the NMR structure of the KRAB-A box from a mouse KRAB-ZFP (UniProt A0A087WRJ1; Saito *et al*, 2003), we were able to build an initial atomic model of the ZNF93 KRAB-A box bound to KAP1 (Fig 1C and D). To validate this model, we introduced methionine point mutations throughout the KRAB domain, generating the ZNF93 variants I11M, C20M, L28M and L40M. The corresponding residues in the ZNF10 KRAB domain were previously shown to tolerate mutation to methionine without loss of silencing function (Tycko *et al*, 2020). Selenomethionine derivatives of these four ZNF93 mutants were purified in complex with KAP1 RBCC and crystallized. X-ray diffraction data were collected (at the selenium K absorption edge) and anomalous Fourier maps calculated. For each mutant, the position of the newly introduced selenium site was successfully located in the anomalous maps, allowing unambiguous identification of the mutated residue and confident sequence assignment (Figs 1E and EV1).

Structure of a KRAB domain in complex with KAP1

The structure contains one KAP1 RBCC dimer, two SMARCAD1 CUE1 domains, and one ZNF93 KRAB domain in the crystallographic asymmetric unit (Fig 2A and B). Consistent with previous biochemical studies (Stoll *et al*, 2019), a single KRAB domain binds the KAP1 RBCC dimer, near the twofold axis, in the central region of the coiled-coil domain. Contacts with KAP1 are exclusively mediated by the KRAB-A box (residues 3–44 of ZNF93), while the KRAB-

 © 2022 The Authors

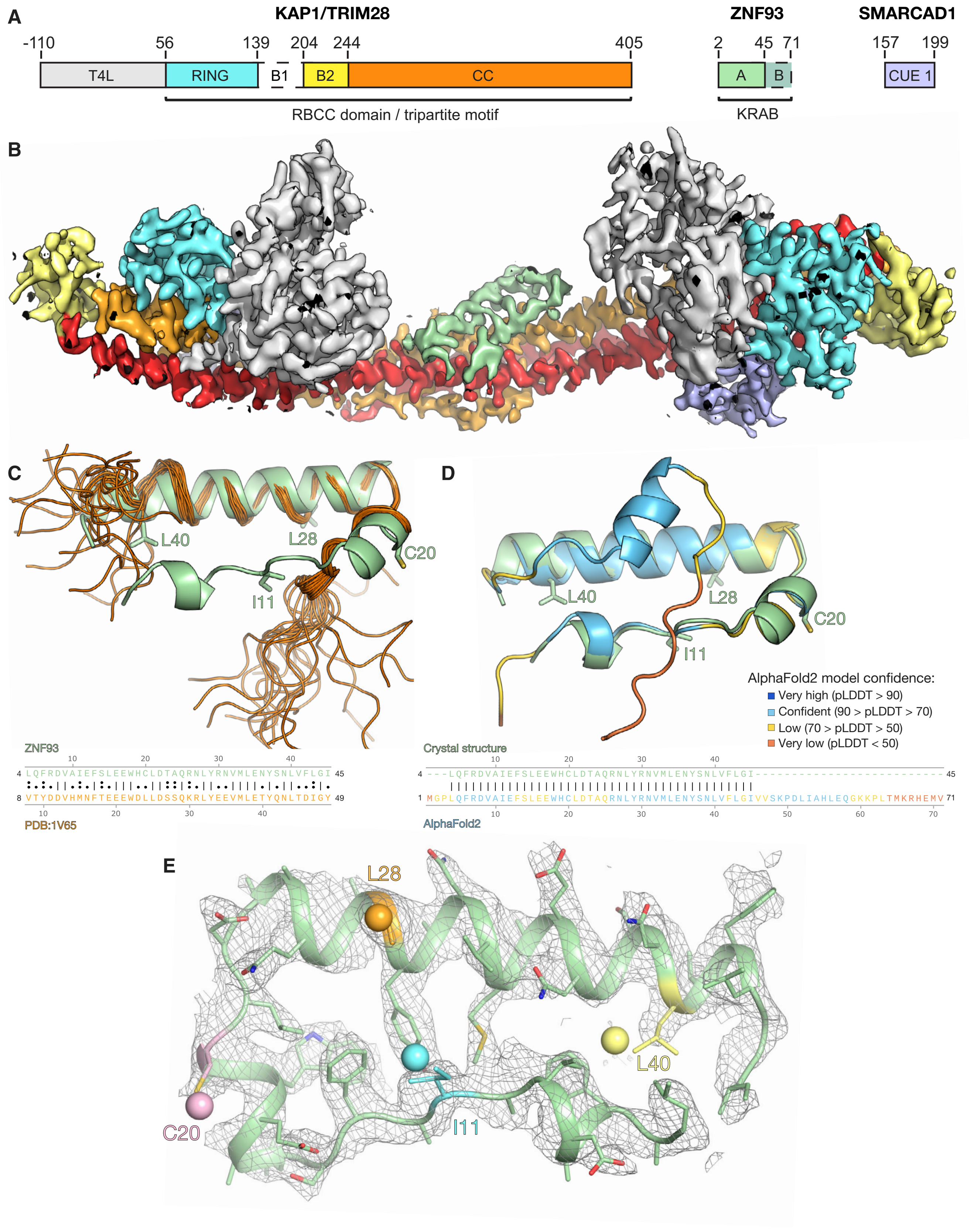
A
KAP1/TRIM28
-110
56
139
204
244
405
T4L
RING
B1
B2
CC
RBCC domain / tripartite motif
ZNF93
2
45
71
A
B
KRAB
SMARCAD1
157
199
CUE 1
B
C
L40
L28
C20
I11
ZNF93
4 LQFRDVAIEFSLEEWHCLDTAQRNLYRNVMLENYSNLVFLGI 45
8 VTYDDVHMNFTEEEWDLLDSSQKRLYEEVMLETYQNLTDIGY 49
PDB:1V65
D
L40
L28
C20
I11
AlphaFold2 model confidence:
Very high (pLDDT > 90)
Confident (90 > pLDDT > 70)
Low (70 > pLDDT > 50)
Very low (pLDDT < 50)
Crystal structure
4 ---LQFRDVAIEFSLEEWHCLDTAQRNLYRNVMLENYSNLVFLGI---------------------------- 45
1 MGPLQFRDVAIEFSLEEWHCLDTAQRNLYRNVMLENYSNLVFLGIVVSKPDLIAHLEQGKKPLTMKRHEMV 71
AlphaFold2
E
L28
L40
C20
I11

Figure 1.

◀ **Figure 1. Structure determination of the ZNF93-KAP1 core complex. See also Fig EV1.**

A Domain organization of the crystallized complex. T4L, T4 lysozyme; B1, B-box 1; B2, B-box 2; CC, coiled-coil.
B $2F_o - F_c$ electron density map for one asymmetric unit. The map is contoured at 1 σ and colored by domain as in (*A*), except the second CC, which is in red.
C Superposition of the crystal structure of ZNF93 KRAB-A (green) on the solution NMR structure of the KRAB-A box from a mouse KRAB-ZFP (UniProt A0A087WRJ1; orange; Saito *et al*, 2003). A sequence alignment of the two domains is shown below.
D Superposition of the ZNF93 KRAB-A crystal structure (green) and the AlphaFold2 prediction (Jumper *et al*, 2021; Varadi *et al*, 2022) of ZNF93 KRAB (colored by confidence score, pLDDT). A sequence alignment of the two domains is shown below.
E Close-up of the electron density ($2F_o - F_c$ map) for the wild-type ZNF93 KRAB-A domain, contoured at 1.0 σ. Residues chosen for mutation to methionine are highlighted in cyan (I11), pink (C20), orange (L28) or yellow (L40). The selenium sites located for each of these variants in anomalous Fourier maps are shown as spheres.

Table 1. Crystallographic data collection, refinement, and structure validation parameters and statistics for complexes containing ZNF93 KRAB, T4 lysozyme fused KAP1 RBCC(ΔB1), and SMARCAD1 CUE1. See also Figs 1 and EV1.

	Wild-type native	ZNF93 I11M, SeMet	ZNF93 C20M, SeMet	ZNF93 L28M, SeMet	ZNF93 L40M, SeMet
**Data collection**					
Space group	*C* 1 2 1	*C* 1 2 1	*C* 1 2 1	*C* 1 2 1	*C* 1 2 1
Cell dimensions					
a, b, c (Å)	190.3, 68.6, 149.1	186.8, 69.9, 156.1	187.2, 69.8, 156.9	187.3, 70.0, 156.8	187.9, 69.8, 157.7
α, β, γ (°)	90, 114.0, 90	90, 113.6, 90	90, 113.8, 90	90, 113.8, 90	90, 113.9, 90
Wavelength (Å)	0.9795	0.97949	0.9795	0.97951	0.9795
Resolution (Å)[a]	61–2.8 (2.9–2.8)	52–3.5 (3.63–3.5)	52–3.3 (3.42–3.3)	51–3.5 (3.63–3.5)	63–3.5 (3.63–3.5)
Observations[a]	119,389 (12,179)	91,722 (8,800)	10,8,241 (10,531)	91,049 (8,870)	90,349 (8,968)
Unique reflections[a]	42,537 (4,206)	23,605 (2,323)	28,280 (2,786)	23,798 (2,324)	23,851 (2,347)
R_{merge}[a]	0.0747 (0.50)	0.129 (0.67)	0.126 (1.0)	0.157 (1.0)	0.115 (0.73)
R_{pim}[a]	0.0518 (0.34)	0.0751 (0.39)	0.0743 (0.61)	0.0917 (0.60)	0.0681 (0.43)
$\langle I \rangle / \sigma I$[a]	21 (2.9)	13 (1.8)	14 (1.8)	12 (1.8)	9 (1.9)
Completeness (%)[a]	97.3 (97.3)	99.2 (99.0)	99.5 (98.9)	99.3 (98.7)	99.2 (99.5)
Multiplicity[a]	2.8 (2.9)	3.9 (3.8)	3.8 (3.8)	3.8 (3.8)	3.8 (3.8)
CC(1/2)[a]	0.99 (0.70)	0.99 (0.87)	0.99 (0.58)	0.97 (0.68)	0.98 (0.87)
**Refinement**					
R_{work}/R_{free}	0.2255/0.2728	0.2288/0.2696	0.2547/0.3083	0.2571/0.3051	0.2430/0.2941
Number of non-H atoms					
Protein	7,889	8,167	8,133	8,163	8,213
Zn^{2+} Ions	8	8	8	8	8
Solvent	0	0	0	0	0
Mean B-factor (Å^2)	69	141	135	156	152
Clashscore[b]	8.98	2.44	2.45	3.11	3.40
RMS deviations					
Bond lengths (Å)	0.013	0.003	0.003	0.003	0.003
Bond angles (°)	1.6	0.68	0.71	0.85	0.73
Ramachandran plot					
% favored	97.2	96.5	96.6	95.8	96.5
% allowed	2.3	2.9	3.0	3.7	2.8
% outliers	0.5	0.6	0.4	0.5	0.7
PDB code	7Z36				

[a]Numbers in parentheses refer to the highest resolution shell.
[b]Clashscore was calculated with Phenix v1.20 (Adams *et al*, 2011).

B box (residues 45–71) is disordered and not visible in the electron density map. This is consistent with previous observations that the KRAB-A box is sufficient for KAP1 binding and transcriptional repression, whereas KRAB-B has an accessory function and is absent in some KRAB-ZFPs (Margolin *et al*, 1994; Witzgall *et al*, 1994; Peng *et al*, 2007; Tycko *et al*, 2020). The KRAB-A box

 © 2022 The Authors

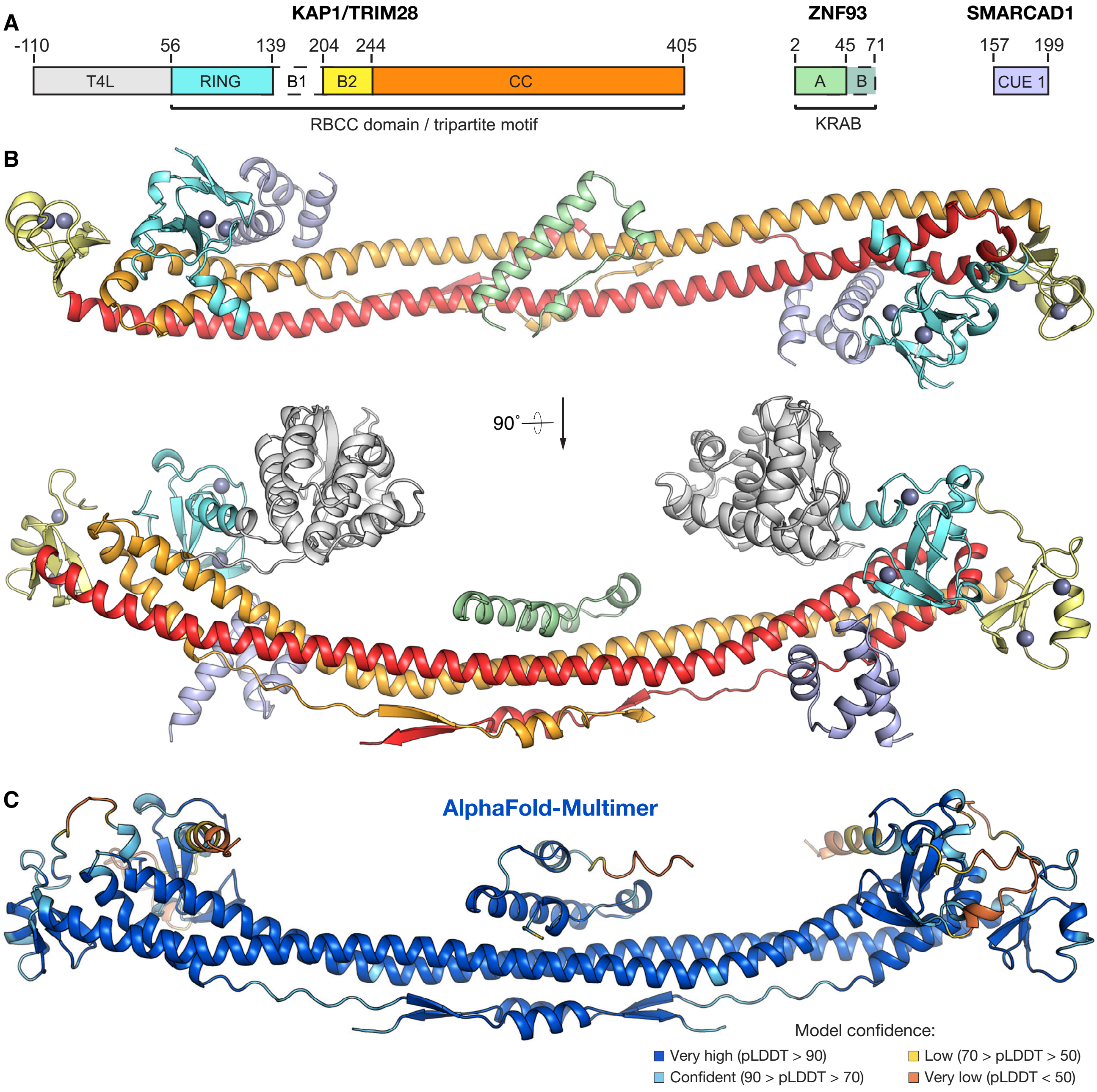

Figure 2. Structure of the ZNF93 KRAB domain of bound to KAP1 RBCC.

A Domain organization of the crystallized complex (as in Fig 1A).

B Overall structure of the KRAB-KAP1 complex. Domains are colored as in *(A)*. Zn atoms are shown as gray spheres.

C Structure of ZNF93 KRAB:KAP1 RBCCΔB1 complex predicted by AlphaFold-Multimer (Evans *et al*, 2021; Mirdita *et al*, 2022).

backbone is U-shaped and contains three α-helical segments: a single helical turn at the N-terminus (α1, residues 6–9), a short central helix (α2, residues 15–20) and a longer C-terminal helix (α3, residues 23–43). The overall conformation of the KRAB-A box in the refined crystal structure remains similar to the AlphaFold2 model of the ZNF93 KRAB and the KRAB-A box NMR structure that guided model building (Fig 1A and B). The AlphaFold2 model spans the entire KRAB domain, including a two-turn helix and random coil for the KRAB-B box, but the confidence score for most of the KRAB-B box is low (pLDDT < 70). In the NMR structure, the N- and C-termini of the KRAB-A box and the entire KRAB-B box are unstructured and highly flexible, consistent with biophysical data showing that KRAB domains are largely unstructured in isolation (Peng *et al*, 2007). We conclude that KRAB domains are mostly disordered

prior to binding KAP1 (with some α-helical character in the KRAB-A box), and that the N- and C-termini of the KRAB-A box adopt a conserved, largely α-helical fold upon binding KAP1.

The RBCC domain of KAP1 in contrast displays no significant conformational changes in response to KRAB binding. The RING, B-box 2, and bound SMARCAD CUE1 domains are all distal from the KRAB-KAP1 interaction interface and unaffected by KRAB binding. The KAP1 coiled-coil domain has a higher curvature in the KRAB-KAP1 complex structure than in previously determined crystal structures of the KAP1 RBCC domain. However, the curvature of the coiled-coil domain varied in different crystal structures of KAP1 RBCC, demonstrating a degree of flexibility. Hence, it is unclear to what extent the crystal packing or KRAB binding contribute to the increased coiled-coil curvature.

After we completed and refined the crystal structure, AlphaFold-Multimer became available (preprint: Evans *et al*, 2021; Mirdita *et al*, 2022). We used it to predict the structure of a 1:2 ZNF93 KRAB:KAP1 RBCC complex. The resulting model was remarkably similar to our crystal structure (Fig 2C; Rmsd 3.1 Å), providing mutual validation of the two models.

Key interacting residues in KRABs and KAP1 are highly conserved

KRAB domains bind to KAP1 with high affinity, with dissociation constants in the low nanomolar range (Stoll *et al*, 2019). Our structure of ZNF93 KRAB bound to KAP1 reveals that the interaction is mostly hydrophobic in nature, with a buried surface area of 1,108 Å^2. The KAP1-binding surface of the KRAB domain is enriched in exposed hydrophobic amino acids and recognizes a hydrophobic patch in the central region of the KAP1 coiled-coil domain (Fig 3A). The hydrophobic core of the KRAB-KAP1 interface is surrounded by a network of polar interactions and salt bridges (Fig 3A). The KRAB forms contacts with both subunits of the KAP1 dimer. Hence, despite having an essentially identical structure, each KAP1 subunit binds to different regions of the KRAB (Fig 3).

The amino acid sequences of KRAB-ZFP KRAB-A boxes are highly conserved across tetrapod vertebrates, except for a small KRAB-ZFP subset, which is thought to have acquired KAP1-independent functions (Figs 3B and EV2; Helleboid *et al*, 2019). Fused to catalytically inactive Cas9, the KRAB domains of ZNF10 and ZIM3 allow potent gene repression that can be programmed in a sequence-specific manner via the CRISPRi approach (Margolin *et al*, 1994; Gilbert *et al*, 2014; Thakore *et al*, 2015; Alerasool *et al*, 2020). Deep mutagenesis studies of ZNF10 have identified the residues essential for KRAB repressor function (Tycko *et al*, 2020). These residues are generally conserved in the KRAB-ZFP family and across species. Our structure of the KRAB-KAP1 complex provides a mechanistic explanation for these observations. Mutation of the highly conserved Asp8 and Val9 residues in the KRAB-A box results in loss of silencing; both residues form direct contacts with KAP1 in the KRAB-KAP1 structure, with Val9 buried in the hydrophobic core and Asp8 forming a salt bridge with Arg304 of KAP1 (Fig 3C). As noted above, hydrophobic contacts form the core of the KRAB-KAP1 interface. Besides Val9, these hydrophobic contacts involve Ile11, Phe13, Leu21, Tyr29, Val32, and Met33 in the KRAB domain, all of which are conserved and required for silencing (Tycko *et al*, 2020). Phe42 forms weak hydrophobic contacts with several neighboring residues, both in KAP1 and within the KRAB. The ZNF10 KRAB, used in first-generation CRISPRi, has a serine in the equivalent position (Ser51), and mutation of this serine to phenylalanine increases repression activity (Tycko *et al*, 2020). Notably, the KRAB domain with the highest reported repression potency in CRISPRi, the ZIM3 KRAB (Alerasool *et al*, 2020), also has a serine at this position (Ser46; Fig EV2), suggesting that the potency of ZIM3 could be further increased by the substitution S46F.

Among the residues forming the ring of polar interface contacts, mutation of Glu17, one of the most conserved residues in the KRAB domain, to any other amino acid results abolishes KAP1 silencing (Tycko *et al*, 2020). This key KRAB domain residue forms a salt bridge with Lys296 and a hydrogen bond with Gln292 of KAP1 (Fig 3C). Similarly, Glu35 and Asn36, which are also essential for silencing, form polar contacts with residues in the KAP1 coiled-coil domain (Fig 3C), including a salt bridge between Glu35 and Arg311 in KAP1. To assess the contribution of this salt bridge to KRAB1-KAP1 complex formation, we generated the charge reversal mutants ZNF93 E35R and KAP1 R311E and measured the binding constants of resulting KRAB-KAP1 complexes by surface plasmon resonance (SPR). The mutation E35R in ZNF93 KRAB reduced its binding affinity for wild-type KAP1 26-fold (from a K_d of 7.5 nM to 198 nM; Figs 4A and EV3). Combining the ZNF93 KRAB E35R and KAP1 R311E variants, which restored charge complementarity, partially restored binding affinity (Fig 4A).

Trp18 is an outlier in that it is highly conserved (Figs 3 and EV2) and required for silencing (Tycko *et al*, 2020) but does not contribute to the KAP1 binding interface or appear to stabilize the KRAB domain fold (Fig 3C). The C-terminal KRAB-A sequence is more variable, but a strong preference for aliphatic residues at positions 40 and 43 is explained by the structure as these residues form part of the hydrophobic core of the KRAB-KAP1 interface. Charged side chains at these positions disrupt silencing (Tycko *et al*, 2020).

Mutations in the KRAB-KAP1 interface abolish repression of L1 and SVA reporters

Our KRAB-KAP1 structure identifies the complete set of residues that form contacts at the binding interface. To assess the importance

Figure 3. Molecular details of the KRAB-KAP1 interface. See also Fig EV2.

A The KRAB-KAP1 complex is shown in surface representation, with the KRAB domain in its natural orientation (*top*) or rotated by 180° to reveal the interaction surface contacting KAP1 (*bottom*). Residues in the interface are colored according to their atomic properties using the YRB scheme (Hagemans *et al*, 2015).

B KRAB domain Hidden Markov Model (HMM) logo (Mistry *et al*, 2021). Residue numbers below the logo refer to the KRAB-A consensus sequence (to convert to ZNF93 residue numbers, add 2).

C Closeups of key residues in the KRAB-KAP1 interface. The corresponding positions in the KRAB consensus sequence are shown in the lower panels (logo residue numbers refer to the consensus sequence; add 2 to obtain ZNF93 sequence). 12 KRAB residues required for KAP1 binding (Tycko *et al*, 2020) are highlighted in the logos in cyan. Aliphatic residues forming part of the hydrophobic core of the KRAB-KAP1 interface are highlighted in yellow.

 © 2022 The Authors

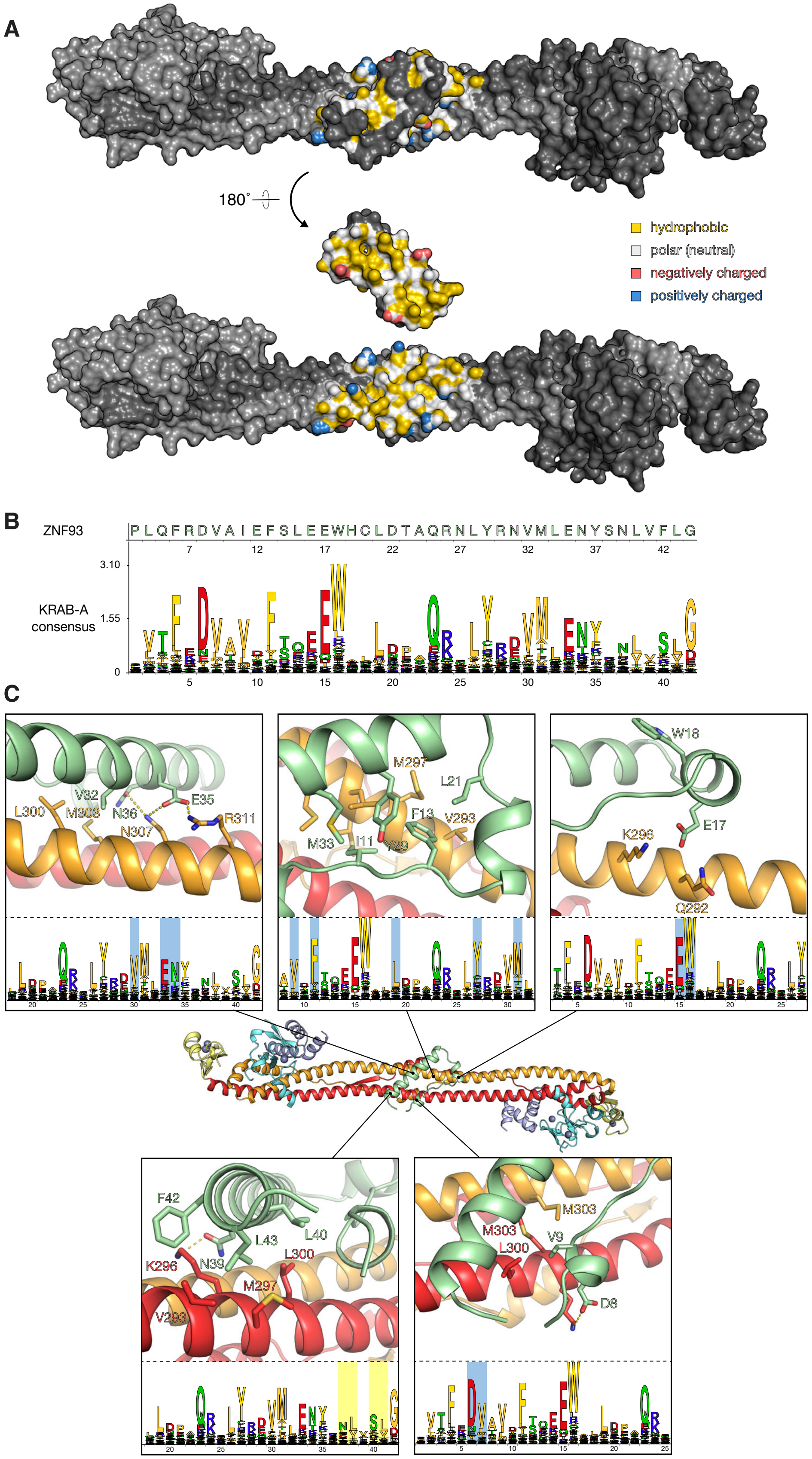
A
180°
hydrophobic
polar (neutral)
negatively charged
positively charged
B
ZNF93
P L Q F R D V A I E F S L E E W H C L D T A Q R N L Y R N V M L E N Y S N L V F L G
KRAB-A consensus
3.10
1.55
0
C
V32
E35
L300
M303
N36
R311
N307
M297
L21
F13
V293
M33
I11
Y29
W18
E17
K296
Q292
F42
L43
L40
K296
N39
L300
M297
V293
M303
M303
L300
V9
D8

Figure 3.

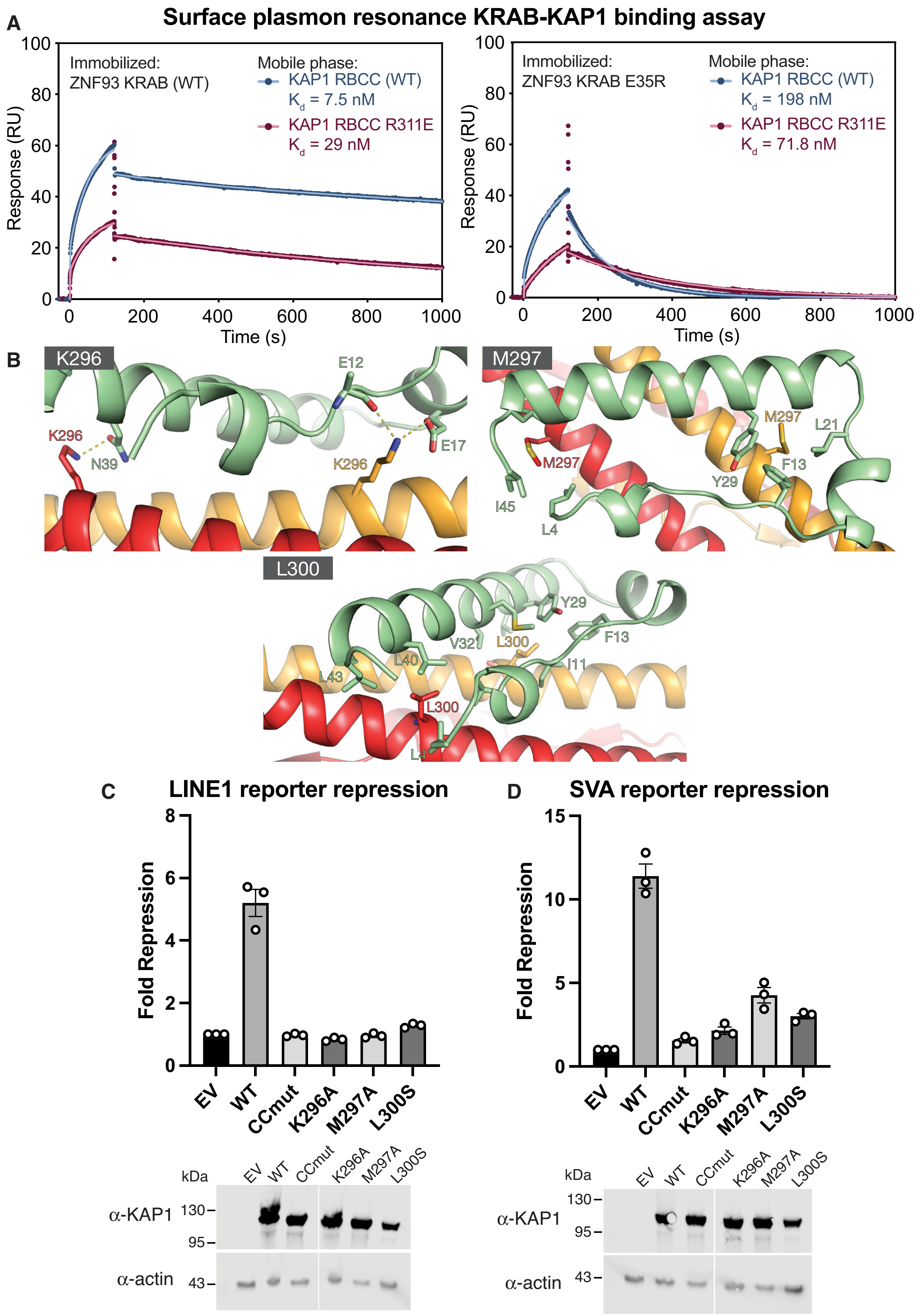

Figure 4.

© 2022 The Authors

Figure 4. Effects of point mutations in the KRAB binding site and KAP1 binding and silencing. See also Fig EV3.

A Surface plasmon resonance (SPR) KAP1-KRAB binding assay. MBP-KRAB was immobilized on the chip. WT or R311E KAP1 RBCC were flowed over the chip. Data points are shown in dark red or blue; fits are shown as light red or blue lines. See Fig EV3 for binding kinetics constants.

B Position of the mutations in the KRAB-KAP1 interface.

C LINE-1 reporter repression with single point mutants and a previously described KRAB binding-deficient KAP1 variant containing four mutations in the CC domain (CCmut; V293S/K296A/M297A/L300S).

D SVA reporter repression with the same set of mutants as in *(B)*. Data were normalized to KAP1 KO cells transfected with an empty vector (EV).

Data information: In (C) and (D), data are presented as fold-repression of reporter luciferase luminescence. Error bars represent standard error of the mean between measurements ($n = 3$). Data are representative of at least three independent (biological replicate) experiments. Lower panels: Western blots of cell lysates from KAP1 KO HEK293T cells transfected with each of the variants or empty vector. Uncropped blots available in Source Data.

Source data are available online for this figure.

of individual key contacts, we measured the effects of structure-based interface mutations on KRAB-dependent transcriptional silencing in a dual luciferase reporter assay. We used previously described reporter constructs in which sequences from LINE-1 element repressed by ZNF93 or an SVA-D element repressed by ZNF91 were cloned upstream of firefly luciferase (Jacobs *et al*, 2014; Robbez-Masson *et al*, 2018). KAP1-knockout (KO) HEK 293T cells were cotransfected with the reporter plasmid and plasmids encoding ZNF93 or ZNF91, WT or mutant KAP1, and *Renilla* luciferase under a constitutive promoter. If WT KAP1 was present, the LINE-1 and SVA reporters were both efficiently silenced (Fig 4C and D). Mutation of Lys296 in the KAP1 coiled-coil domain, which is involved in multiple electrostatic interactions with the KRAB domain (Fig 4B), completely abolished repression of both reporters. Similarly, mutation of Met297 or Leu300, which form part of the hydrophobic core of the KRAB-KAP1 interface, to serine resulted in loss of silencing (Fig 4C and D). We previously showed that the KAP1 variant CCmut, containing four mutations in the central region of the CC domain (V293S/K296A/M297A/L300S), is deficient in KRAB binding and transcriptional repression (Stoll *et al*, 2019). The data presented indicate that Leu296, Met297, and Leu300 are each required for KRAB binding and repression by KAP1.

Disruption of KAP1-KRAB binding affects KAP1 recruitment and H3K9 trimethylation

To assess the importance of KRAB binding residues in KAP1 recruitment to chromatin genome-wide, we mapped KAP1 distribution by chromatin immunoprecipitation sequencing (ChIP-seq) in KAP1 KO cells complemented with WT or KRAB-binding-deficient (CCmut) KAP1. We found that KAP1 enrichment in the WT-complemented cells occurred at two types of loci: H3K9me3-positive regions and gene promoters (Fig EV4A and B). No KAP1 enrichment was observed over H3K9me3-positive regions in the CCmut-complemented cells, indicating that KAP1 recruitment to these sites depends on KRAB binding. By contrast, KAP1 remained enriched over gene promoters in the CCmut-complemented cells, suggesting that KAP1 recruitment to promoters does not depend on KRAB binding (Fig EV4A). Notably, KAP1-promoter interactions involving the PHD-bromodomain region of KAP1 have been reported previously (Bacon *et al*, 2020). We cannot, however, discount the possibility that the promoter peaks are an artifact of overexpression and crosslinking of the recombinant protein to open chromatin (relative expression levels in WT cells and KAP1-complemented KAP1 KO cells are shown in Fig EV4C and D).

While providing support for the model KAP1 is recruited to specific genomic loci via KRAB-ZFP binding, signal enrichment in KAP1 ChIP-seq experiments was small relative to the background, limiting the scope of our analysis. Deposition of the repressive epigenetic mark H3K9me3 by SETDB1 is an essential component of targeted KAP1-dependent silencing by KRAB-ZFPs and via CRISPRi (Schultz *et al*, 2002; Thakore *et al*, 2015). Immunoprecipitation experiments showed that WT and CCmut KAP1 recruit SETDB1 equally well, in a SUMO-dependent manner (Fig EV4E), demonstrating that SETDB1 recruitment by KAP1 is independent of KRAB binding. To assess the importance of KAP1-KRAB binding in genome-wide H3K9-trimethylation, we measured the distribution of H3K9me3 in cells expressing different KAP1 variants with the CUT&RUN epigenomic profiling method (Skene *et al*, 2018). We observed a massive loss of H3K9me3 genome-wide in KAP1 KO cells, representing at least 65% of mappable H3K9me3 peaks (Figs 5A and B, and EV5). For example, H3K9me3 was significantly depleted over thousands of retrotransposons from all classes, including full-length primate-specific LINE-1 subfamilies, SVAs and LTR elements (Fig 5C and D). The extent of H3K9me3 loss in human cells upon KAP1 depletion is comparable to ChIP-seq data from mouse ESCs (Coluccio *et al*, 2018; Jang *et al*, 2018). Notably, H3K9me3 was more modestly reduced at sites targeted by the HUSH complex, primarily intronic LINE-1 s and long exons (Fig EV5; Douse *et al*, 2020; Seczynska *et al*, 2022). The measurable reduction in H3K9me3 over HUSH-bound loci in KAP1 KO cells may reflect the cooperation between the two complexes at certain loci (Robbez-Masson *et al*, 2018). Complementation of KAP1 KO cells with wild-type KAP1 robustly restored H3K9me3 levels. However, the KRAB binding-deficient KAP1 mutant completely failed to restore H3K9 methylation at KAP1-regulated loci (Figs 5 and EV5) despite being expressed in the nucleus at comparable levels to the WT protein and being able to interact with SETDB1 (Fig EV4C–E). Taken together, the data demonstrate that the KRAB-binding residues of KAP1 are required for targeted KAP1-dependent H3K9 methylation.

Discussion

Here, we report the crystal structure of the core complex between KAP1 and the KRAB domain from a representative KRAB-ZFP, the human LINE-1 repressor ZNF93. KRAB-ZFPs have grown into the largest family of mammalian transcriptional factors, driven by their role in safeguarding the genome from TEs. In parallel, KRAB-ZFPs have acquired vital functions in controlling gene expressing during

© 2022 The Authors

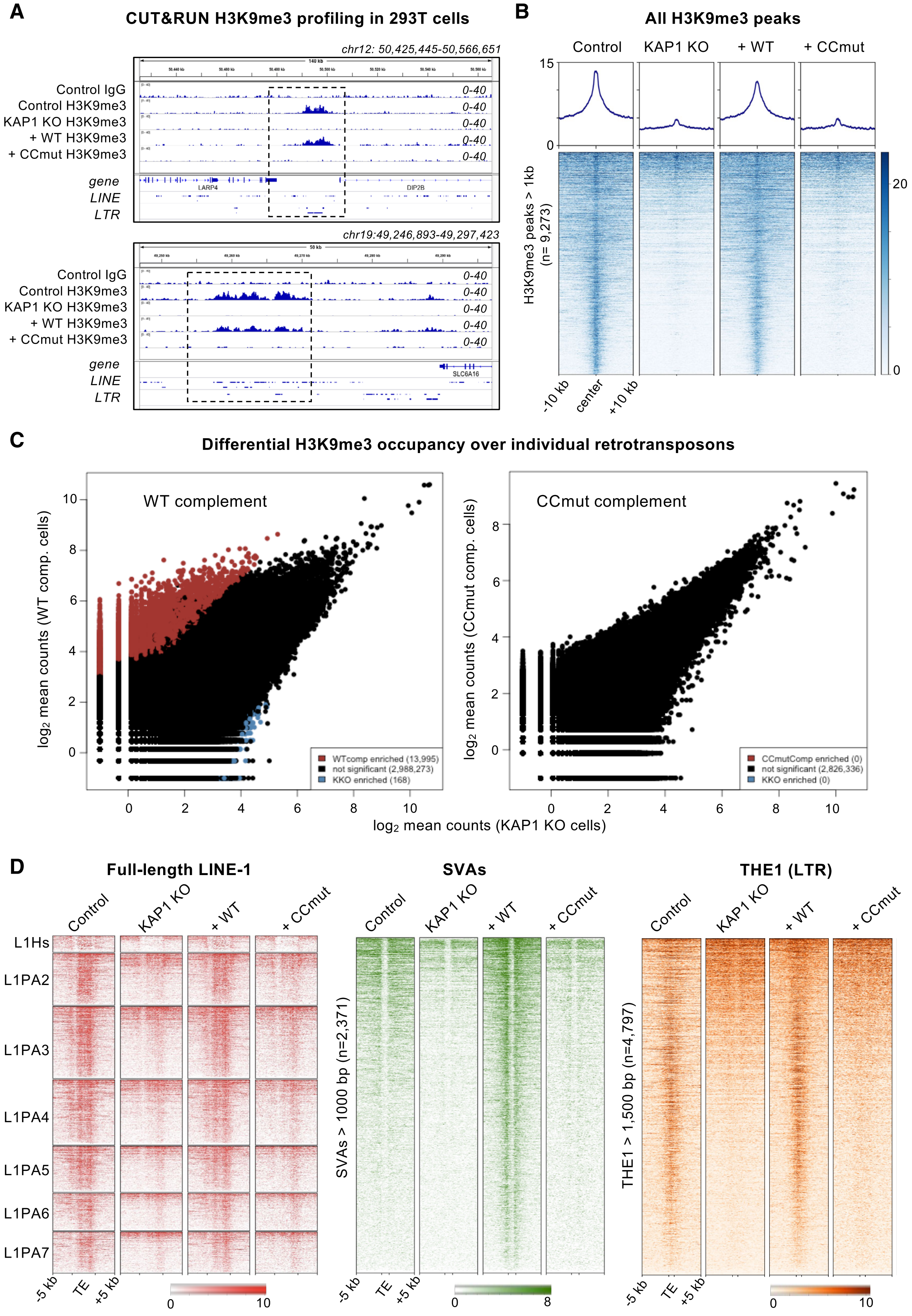

Figure 5.

© 2022 The Authors

Figure 5. Genome-wide CUT&RUN analysis of H3K9me3 distribution in cells expressing wild-type and KRAB binding-deficient variants of KAP1. See also Figs EV4 and EV5.

A Example genome browser snapshots of H3K9me3 distribution over the hg38 reference in the presence of different KAP1 variants. H3K9me3 distribution is shown at a HERVK9-int element (upper) and a cluster of LINE-1 elements (lower). A control IgG track from parent HEK293T cells is shown for comparison. WT, wild-type; CCmut, KRAB binding-deficient KAP1 variant. Only reads mapping uniquely (MAPQ > 10) were retained. Scales are in RPKM, reads per kilobase per million. Experiments were run in duplicate (biological replicates) with similar results.

B Heatmaps and summary plots illustrating H3K9me3 levels over H3K9me3 peaks genome wide, in cells expressing different KAP1 variants. Peaks were called on Control cells using SEACR in stringent mode (Meers *et al*, 2019) against the IgG control, and only regions longer than 1 kb retained. The summary plots illustrate mean values for each sample. Only reads mapping uniquely (MAPQ > 10) were retained. Experiments were run in duplicate with similar results.

C Pairwise quantifications of H3K9me3 CUT&RUN counts for KAP1-complemented cells ($n = 2$) versus KAP1 KO cells ($n = 2$) over RepeatMasker retrotransposons (hg38) generated with DESeq2 (Love *et al*, 2014). Only reads mapping uniquely (MAPQ > 10) were retained. Red, H3K9me3 enriched in complemented cells; blue, H3K9me3 enriched in KAP1 KO cells. Enrichment cutoff: $P < 0.05$; log-fold change ±3.

D Heatmaps showing H3K9me3 CUT&RUN signal enrichment over full-length (> 6 kb) LINE-1 subfamilies (left, red), reference SVAs > 1 kb (center, green) and THE1 LTR elements > 1.5 kb (right, orange) in cells expressing different KAP1 variants. H3K9me3 is rescued upon complementation with WT KAP1 but not CCmut KAP1. Only reads mapping uniquely (MAPQ > 10) were retained; note that only the flanking regions of SVAs are mappable even with a 2 × 150-bp paired end sequencing strategy. Experiments were run in duplicate with similar results.

vertebrate development. A KRAB-dCas9 fusion protein is the key reagent underpinning the CRISPRi technology (Gilbert *et al*, 2014; Thakore *et al*, 2015; Alerasool *et al*, 2020). Our structure provides a detailed and complete three-dimensional atlas of the KRAB-KAP1 binding interface. The functional importance of this interface is validated by our repression reporter assay and epigenomic profiling data.

In the crystal structure, the electron density for the KRAB domain was weaker than for KAP1, potentially indicating some residual degree of conformational flexibility or heterogeneity in the KRAB domain bound to KAP1. Due to the 2:1 stoichiometry of the KAP1:KRAB complex, the KRAB domain could in principle bind to the KAP1 dimer in two equivalent orientations, related by the dimer dyad. However, there is no evidence in the crystal structure of KRAB binding to the KAP1 RBCC dimer in an alternative orientation or conformation (related by the dimer dyad or not). This could be due to steric hindrance from the crystal packing preventing the KRAB from binding in the dyad-related orientation. Alternatively, the KAP1 dimer may have some inherent asymmetry such that a single binding orientation of the KRAB is favored. Supporting the latter, solution biophysics studies showed that full-length KAP1 forms asymmetric dimers that bind HP1 with a 2:1 KAP1:HP1 stoichiometry (Fonti *et al*, 2019) The HP1-binding domain is located in the C-terminal half of KAP1, outside the RBCC (Schultz *et al*, 2002). Together, the available data support the model that the KAP1 dimer has some degree of intrinsic asymmetry, which is functionally important in that it determines the 2:1 stoichiometry of the complexes with KRAB-ZFPs and HP1.

The KRAB-B box of ZNF93 was present in the crystallized construct, but there were no interpretable features in the electron density for the KRAB-B box. AlphaFold2 predicts with medium confidence (pLDDT = 70–90) that the first half of the KRAB-B sequence forms a two-turn helix, which packs against the C-terminal KRAB-A helix, on the opposite side from the KAP1 binding surface (Fig 1). A weak electron density peak was present in the KRAB-KAP1 crystal structure (in the $F_o - F_c$ Fourier difference map), near where this KRAB-B helix would be expected based on the AlphaFold2 prediction. However, the density was too weak to allow a model to be built, indicating that the KRAB-B box is mobile or disordered in the KRAB-KAP1 complex. Consistent with this, systematic mutagenesis of the KRAB-B box had little effect on KAP1 recruitment, with mutations at only one KRAB-B residue (Pro59) showing a weak effect (Tycko *et al*, 2020).

KRAB-KAP1 complexes recruit multiple effectors that modify the epigenetic conformational landscape of chromatin target loci including HP1, SETDB1, NuRD, and SMARCAD1. Except for SMARCAD1, these effectors bind to sites outside the KAP1 RBCC, in the C-terminal half of KAP1. SMARCAD1 is an ATP-dependent chromatin remodeler thought to be important to generate and maintain the necessary chromatin conformation in pluripotent embryonic stem cells (Ding *et al*, 2018). The KRAB-KAP1 crystal structure shows that the KRAB and SMARCAD1 CUE1 domains bind KAP1 RBCC dimers independently, without steric interference (Fig 2B). Hence, the KRAB-binding site on KAP1 is distal from all known effector binding sites on KAP1, meaning that all KAP1 effectors are expected to be recruited to KRAB-ZFP chromatin-binding sites.

By recruiting chromatin-modifying proteins to KRAB-ZFP-binding sites, KAP1 protects the genome from invasion by TEs and plays a key role in regulating gene expression in early development, across tetrapod vertebrate species. The KRAB-KAP1 interaction lies at the nexus of this vital transcriptional control axis. Our work identifies and functionally validates the KRAB-KAP1 molecular interface. The KRAB-KAP1 structure will guide future efforts to further increase the repression potency of KRAB domains in CRISPRi and potentially other applications. The ability to understand and manipulate how and when KAP1 is recruited to its target loci by KRAB-ZFPs, specifically in stem cells or other pluripotent cells, could create new opportunities to direct cell fate determination, for example, in cell therapy applications.

Materials and Methods

Cell lines and bacterial strains

HEK293T cells were a kind gift from Helen Rowe (University College London). Selenomethionine-labeled proteins were expressed in *Escherichia coli* B834(DE3) cells (Merck). All other proteins were expressed in *Escherichia coli* BL21(DE3) cells (New England BioLabs). Cell lines tested negative for mycoplasma but were not recently authenticated.

Expression vectors

A synthetic gene encoding SMARCAD1 CUE1 (residues 151–198; UniProt Q9H4L7) codon optimized for *Escherichia coli* (*E. coli*) was cloned into the first multiple cloning site (MCS) of the pRSFDuet

© 2022 The Authors

plasmid (Novagen), with N-terminal hexahistidine purification (His_6) tag followed by a TEV protease cleavage site. The T4L-RBCC fusion construct was described previously (Stoll *et al*, 2019). To improve protein crystallization the B-box 1 domain (residues 141–202 of KAP1) was deleted, generating the T4L-RBCCΔB1 plasmid. A codon-optimized gene encoding residues 2–71 of ZNF93 (UniProt P35789) was cloned into MCS1 of pCDFDuet, preceded by Twin-StrepII and MBP purification tags and a HRV 3C protease cleavage site.

For expression in mammalian cells, full-length KAP1 (UniProt Q13263) with triple FLAG tag was cloned into pLEXm as described previously (Stoll *et al*, 2019). For lentivirus production, KAP1 was subcloned into pHRSIN. Expression vectors are available upon request.

Protein expression and purification

T4L-RBCCΔB1-ZNF93 KRAB complexes were produced by co-expression in *E. coli* BL21 (DE3) cells (New England BioLabs). Cells were grown at 37°C in 2 × TY medium. When the optical density (OD_{600}) of the cultures reached 0.4–0.5, the culture medium was supplemented with 50 μM $ZnSO_4$ and the incubator temperature was lowered to 16°C. At an OD_{600} of 0.8, protein expression was induced with 0.2 mM isopropyl-β-D- thiogalactopyranoside (IPTG) for 18 h, before the cells were harvested by centrifugation (6,000 *g* for 15 min). The bacteria pellets were resuspended in wash buffer (50 mM Tris pH 8, 0.2 M NaCl, 0.5 mM TCEP) supplemented with 1:10,000 (v/v) Benzonase nuclease (Sigma) and 1 × cOmplete EDTA-free protease inhibitors (Roche) and lysed by sonication. The lysate was clarified by centrifugation (30 min, 40,000 *g*) and applied to a 5-ml StrepTrap HP column (Cytiva) equilibrated in wash buffer. The column was washed with 30 column volumes (CV) of wash buffer before bound proteins were eluted with wash buffer supplemented with 3 mM D-desthiobiotin (Sigma). The protein was transferred into wash buffer without D-desthiobiotin and incubated overnight at 4°C with 1:100 (w/w) HRV 3C protease to remove the Twin-StrepII-MPB tag. Uncleaved protein and free tag were subsequently captured using a StrepTrap HP column, and the sample was further purified by size-exclusion chromatography using a HiLoad (16/600) Superdex 200 pg column (Cytiva) equilibrated in 20 mM HEPES pH 8, 0.5 M NaCl, 0.5 mM TCEP. Selenomethionine labeled proteins were expressed in *E. coli* B834 (DE3) cells (Novagen) and purified like the native protein, except that all buffers contained 1 mM TCEP.

E. coli BL21 (DE3) cells were transformed with the His_6-CUE1 pRSFDuet plasmid and grown at 37°C. At $OD_{600} = 0.8$, the incubator temperature was reduced to 18°C, and protein expression was induced with 0.2 mM IPTG for 18 h. The cells were harvested by centrifugation, resuspended in wash buffer (50 mM Tris pH 8, 0.3 M NaCl, 20 mM imidazole, 0.5 mM TCEP), supplemented with 1:10,000 (v/v) Benzonase nuclease (Sigma) and 1 × cOmplete EDTA-free protease inhibitors (Roche), and lysed by sonication. Insoluble cell debris was removed by centrifugation (30 min, 40,000 *g*), and the supernatant was applied to a 5-ml HisTrap HP column (Cytiva) equilibrated in wash buffer. The column was washed with 30 CV of wash buffer before bound proteins were eluted with elution buffer (50 mM Tris pH 8, 0.3 M NaCl, 250 mM imidazole, 0.5 mM TCEP). The eluted protein was further purified by size-exclusion chromatography using a HiLoad (26/600) Superdex 75 pg column (Cytiva) equilibrated in 20 mM HEPES pH 8, 0.2 M NaCl.

X-ray crystallography

Crystals were grown at 18°C by sitting drop vapor diffusion. Purified T4L-RBCCΔB1-ZNF93 KRAB complex (5 g l^{-1}, 43 μM) was mixed with SMARCAD1 CUE1 domain (5 g l^{-1}, 610 μM) at a 1:2.2 molar ratio. The sample was subsequently mixed with an equal volume of reservoir solution optimized from the Index screen (Hampton Research): 11% (w/v) PEG 5000 MME, 5% Tacsimate, and 0.1 M HEPES pH 7. Crystals appeared after 2 days and were frozen in liquid nitrogen with 20% glycerol as a cryoprotectant. X-ray diffraction data were collected at 100 K at Diamond Light Source (beamline i04) and processed with xia2 (DIALS, AIMLESS). The structure was solved by molecular replacement using T4L-fused KAP1 RBCC (PDB 6QAJ; Stoll *et al*, 2019) as a search model. The atomic models for the SMARCAD1 CUE1 domain (PDB 6QU1; Lim *et al*, 2019) were docked into the phased electron density map. The ZNF93 KRAB domain was built using the Alphafold2 prediction of ZNF93 and the NMR structure of a mouse KRAB domain (PDB 1V65; Saito *et al*, 2003) as guides. The model was iteratively refined using COOT and PHENIX (Adams *et al*, 2011). See Table 1 for data collection and refinement statistics.

Selenomethionine-labeled point mutants crystallized in similar conditions as the native WT complex and were cryoprotected with either 20% glycerol or 25% ethylene glycol prior to freezing in liquid nitrogen. Data was collected at a wavelength of 0.9795 Å. The structures were solved by molecular replacement using the structure of the T4L-RBCCΔB1 - ZNF93 KRAB - CUE1 complex. The models were refined using the LORESTR pipeline (Nicholls *et al*, 2017) implemented in CCP4 (Cowtan *et al*, 2011) and PHENIX. Selenium sites were located using Phaser MR-SAD in PHENIX (McCoy *et al*, 2007).

AlphaFold2 structure predictions

The AlphaFold2 structure prediction for ZNF93 was obtained from the EMBL-EBI AlphaFold Protein Structure Database, accession number P35789 (Jumper *et al*, 2021; Varadi *et al*, 2022). The structure of the ZNF93 KRAB-KAP1 RBCC complex was predicted by submitting the sequences from ZNF93 (amino acids 2–71) and KAP1 (amino acids 50–140 and 203–413) for structure prediction using the Colab implementation of AlphaFold-multimer, specifying a 1:2 stoichiometry (https://colab.research.google.com/github/deepmind/alphafold/blob/main/notebooks/AlphaFold.ipynb).

Transcriptional silencing assay

The transcriptional silencing activity of KAP1 mutants was measured using reporter plasmids in which SVA or LINE-1 sequence upstream of a minimal SV40 promoter enhances firefly luciferase activity unless KAP1 and the cognate KRAB-ZFP (ZNF91 and ZNF93, respectively) are present to repress the reporter. KAP1 KO HEK293T cells in 24-well plates were cotransfected with 20 ng firefly luciferase reporter plasmid, 0.2 μg plasmid encoding ZNF91 or ZNF93, 0.2 μg pLEXm plasmid encoding WT or mutant KAP1, and 0.4 ng plasmid

© 2022 The Authors

encoding Renilla luciferase using FuGENE 6 (Promega). Luciferase activity was measured 48 h post-transfection using the Dual Luciferase assay kit (Promega) and a PHERAstar FSX microplate reader (BMG Labtech). Replicates were performed on separate days. Firefly luciferase values were normalized to *Renilla* luciferase values to control for transfection efficiency. Statistical significance was assessed with an unpaired *t* test (assuming Gaussian distributions, without Welch's correction) with PRISM 9 (GraphPad).

Western blotting

4×10^5 HEK293T cells were lysed in 100 μl of passive lysis buffer (Promega). 10 μl of cell lysates were separated on a NuPAGE 4–12% Bis-Tris polyacrylamide gel (ThermoFisher). The samples were transferred onto nitrocellulose membranes using the iBlot2 Dry Blotting System (ThermoFisher). The membrane was blocked with 5% (w/v) skim milk powder (Sigma-Aldrich) in PBS for 1 h at room temperature before it was incubated overnight at 4°C with primary antibody diluted in PBS-T (PBS with 0.1% Tween-20) containing 5% (w/v) skim milk powder. Rabbit anti-KAP1 antibody (Abcam, cat. no. ab10484, RRID:AB_297223) was diluted 1:10,000; rabbit anti-actin antibody (Abcam, cat. no. ab219733; RRID:AB_219733) was diluted 1:2,000. Subsequently, the membrane was washed four times with PBS-T and incubated with DyLight 800 goat anti-rabbit IgG (Cell Signaling Technology, cat. no. 5151, RRID:AB_10697505) diluted 1:10,000 in PBS-T containing 5% (w/v) skim milk powder. After 30 min at room temperature, the membrane was washed four times with PBS-T, twice with PBS and once with ultrapure water. Blots were imaged using an Odyssey CLx gel scanner (LI-COR Biosciences).

Generation of stable cell lines

Lentivirus was produced by transfecting HEK293T cells in 6-well plates with 0.6 μg pMD2.G, 1.2 μg p8.91, and 1.2 μg KAP1 pHRSIN using FuGENE 6 (Promega). Virus-containing supernatant was collected 48 h post-transfection, filtered through a 0.45 μm PVDF membrane, and used to infect KAP1 KO HEK239T cells. Transduced cells were selected with 200 $\mu g\ ml^{-1}$ hygromycin.

Surface plasmon resonance (SPR)

SPR was performed on a Biacore T200 system with Series S CM5 sensor chips (Cytiva). Reference and sample channels were equilibrated in 20 mM HEPES pH 8.0, 0.5 M NaCl, and 0.5 mM TCEP at 20°C. MBP-KRAB was immobilized onto the sensor chip until a response unit (RU) value of approximately 300 (ZNF93 WT) or 600 (ZNF93 E35R) was reached. Analytes in 1:2 dilution series at an initial concentration of 40 μM were injected for 120 s followed by a 900 s dissociation phase. After each injection cycle, the sensor surface was regenerated with 20 mM NaOH for 30 s with a 120-s post-regeneration stabilization period. Data were fitted using a biphasic kinetic model with PRISM 9 (GraphPad) to determine k_{on}, k_{off}, and K_d.

Chromatin immunoprecipitation sequencing (ChIP-seq) for KAP1

For each HEK293T cell sample, 10^7 cells grown in 10-cm plates were fixed for 15 min at room temperature with 1% formaldehyde and added directly to the DMEM media (GIBCO). The 10× formaldehyde stock solution was freshly prepared with molecular biology-grade reagents and contained 11% formaldehyde (Sigma-Aldrich, cat. no. F-8775), 50 mM HEPES pH 7.9, 0.1 M NaCl, and 1 mM EDTA. Fixation was quenched by adding 120 mM glycine (Sigma cat. no. G-7403) from a 20× stock solution and incubating for 5 min at room temperature. The cells were washed twice by centrifugation (800 *g*, 4°C, 10 min) and resuspension in PBS, 0.5% IGEPAL CA-630 (Sigma-Aldrich, cat. no. I-8896), 0.1 mM phenylmethylsulfonyl fluoride (PMSF). Cell pellets were then frozen and shipped in dry ice to Active Motif for chromatin preparation, immunoprecipitation with anti-KAP1 antibody (Abcam cat. no. ab10483 RRID:AB_297222), DNA library generation, and sequencing using a NextSeq 500 system (Illumina). Sequencing of the ChIP samples was performed in duplicate alongside a pooled input, with a depth of approximately 30 million reads per sample. Single-end reads were mapped to the hg38 reference using Bowtie2 (—-phred33–very sensitive) and converted to bam files with samtools. Only reads with unique mapping (MAPQ > 10) were retained for analysis. KAP1 occupancy over different genomic regions was assessed with deepTools (computeMatrix and plotHeatmap commands on RPKM-normalized bigwig coverage files) following arithmetic subtraction of input signal (bigwigCompare), and coverage tracks were viewed in IGV.

Immunofluorescence microscopy

Cells were grown in 24-well plates on poly-L-lysine coated coverslips, fixed with 4% formaldehyde in PBS for 15 min and permeabilized with 0.1% Triton X-100 in PBS. After blocking with PBS and 10% FBS for 1 h, samples were incubated for 1 h with the primary antibody (mouse anti-KAP1, Proteintech, cat. no. 66630-1-Ig, RRID: AB_2732886) diluted 1:500 in PBS and 10% FBS. The cells were washed three times with PBS and 10% FBS and then incubated for 1 h with the secondary antibody (Alexa Fluor 488 goat antimouse, ThermoFisher, cat. no. A-11001, RRID:AB_2534069) diluted 1:500 in PBS and 10% FBS. After three washes with PBS and 10% FBS, one wash with PBS, and one wash with water, the coverslips were mounted with ProLong Gold Antifade Mountant with DAPI (ThermoFisher). Images were acquired using a Zeiss LSM 780 confocal microscope with a 63×/1.4 NA oil immersion objective.

Coimmunoprecipitation

1×10^7 HEK293T KAP1 KO cells complemented with 3× FLAG-KAP1 (WT or CCmut) were washed twice with ice-cold PBS. The cell pellets were resuspended in 1 ml of ice-cold lysis buffer containing 50 mM HEPES pH 7.0, 150 mM NaCl, 1% NP-40, 1:100 (v/v) benzonase (Sigma-Aldrich), cOmplete protease inhibitors (Roche), 1 mM PMSF, with or without 20 mM N-ethylmaleimide to preserve SUMO modification. After 30 min at 4°C, insoluble cell debris were removed by centrifugation at 14,000 *g* for 10 min. A 30-μl aliquot was saved as input sample, the rest of the supernatant was incubated with 20 μl of ANTI-FLAG M2 affinity gel (Sigma-Aldrich) equilibrated in wash buffer (50 mM HEPES pH 7.0, 150 mM NaCl, 1% NP-40, cOmplete protease inhibitors). After 2 h at 4°C, the beads were washed twice with 1 ml of wash buffer before bound proteins were eluted with 45 μl of 2× SDS-PAGE sample buffer. 10 μl of each sample were analyzed by western blotting using anti-

© 2022 The Authors

KAP1 antibody (Abcam, cat. no. ab10484, RRID:AB_297223, diluted 1:5,000) and anti-SETDB1 antibody (Proteintech, cat. no. 11231-1-AP, diluted 1:300).

CUT&RUN H3K9me3 profiling

We followed the protocol detailed by Henikoff and colleagues (Skene *et al*, 2018). Briefly, 250,000 cells (per antibody/cell line combination) were washed twice (20 mM HEPES pH 7.5, 0.15 M NaCl, 0.5 mM spermidine, 1× Roche complete protease inhibitors) and attached to ConA-coated magnetic beads (Bangs Laboratories) preactivated in binding buffer (20 mM HEPES pH 7.9, 10 mM KCl, 1 mM $CaCl_2$, 1 mM $MnCl_2$). Cells bound to the beads were resuspended in 50 µl buffer (20 mM HEPES pH 7.5, 0.15 M NaCl, 0.5 mM Spermidine, 1× Roche complete protease inhibitors, 0.02% w/v digitonin, 2 mM EDTA) containing rabbit anti-H3K9me3 (Abcam cat. no. ab8898, RRID:AB_306848) or guinea pig anti-rabbit IgG (American Research Products, cat. no. CSB-PA00150E1Gp) at 1:100 dilution. Incubation proceeded at 4°C overnight with gentle shaking. Tubes were placed on a magnet stand to remove unbound antibody and washed three times with 1 ml digitonin buffer (20 mM HEPES pH 7.5, 0.15 M NaCl, 0.5 mM Spermidine, 1× Roche complete protease inhibitors, 0.02% digitonin). pA-MNase (35 ng per tube, a generous gift from Steve Henikoff) was added in 50 µl digitonin buffer and incubated with the bead-bound cells at 4°C for 1 h. Beads were washed twice, resuspended in 100 µl digitonin buffer, and chilled on ice. Genome cleavage was stimulated by addition of 2 mM $CaCl_2$, briefly vortexed, and incubated on ice for 30 min. The reaction was quenched by addition of 100 µl 2× stop buffer (0.35 M NaCl, 20 mM EDTA, 4 mM EGTA, 0.02% digitonin, 50 ng/µl glycogen, 50 ng/µl RNase A, 10 fg/µl yeast spike-in DNA (a generous gift from Steve Henikoff)) and vortexing. After 10 min incubation at 37°C to release genomic fragments, cells and beads were pelleted by centrifugation (16,000 *g*, 5 min, 4°C) and fragments from the supernatant purified with a nucleospin PCR clean-up kit (Macherey-Nagel). Illumina sequencing libraries were prepared using the Hyperprep kit (KAPA) with unique dual-indexed adapters (KAPA), pooled and sequenced on a NovaSeq6000 (replicate 1) or NextSeq 2,500 (replicate 2) instrument. Paired-end reads (2 × 150) were aligned to the human genome (hg38) using Bowtie2 (—-local–very-sensitive-local–no-mixed–no-discordant–phred33 -I 10 -X 700) and converted to bam files with samtools. Only reads with unique mapping (MAPQ > 10) were retained for all analyses. Peaks relative to the IgG negative control were called on control cells using SEACR in stringent mode (Meers *et al*, 2019). Differential H3K9me3 occupancy was assessed qualitatively with deepTools (computeMatrix and plotHeatmap commands on RPKM-normalized bigwig coverage files) and quantitatively with a combination of featureCounts, HOMER (Heinz *et al*, 2010) and DESeq2 (Love *et al*, 2014). Retrotransposon annotations were downloaded from the RepeatMasker hg38 database. Numerical analysis (e.g. for comparing H3K9me3 changes over KAP1-dependent and HUSH-bound loci) was carried out in R. Intersects between genomic loci were analyzed with bedtools intersect. Two independent CUT&RUN experiments were performed. See https://github.com/NinoPandiloski for code and pipelines used in epigenomic analyses.

Quantification and statistical analysis

No statistical methods were used to predetermine sample size, experiments were not randomized, and the investigators were not blinded to experimental outcomes. Luciferase-reporter cell signaling data are represented as the mean ± standard error of the mean of three replicates (n = 3) conducted in a single independent experiment. Data are representative of at least three independent experiments. Two independent replicates were generated for all genome profiling experiments (CUT&RUN and ChIP-seq).

Data availability

Atomic coordinates: Protein Data Bank 7Z36, DOI:10.2210/pdb7Z36/pdb (http://identifiers.org/PDB/7Z36). X-ray diffraction images: SBGrid Data Bank 880, DOI:10.15785/SBGRID/880 (http://data.sbgrid.org/dataset/880). CUT&RUN and ChIP-seq data: Gene Expression Omnibus GSE215016 (http://www.ncbi.nlm.nih.gov/geo/query/acc.cgi?acc=GSE215016). Uncropped gels are available in the source data files that accompany this publication. Other data are available from the corresponding author upon request. Code and pipelines used in epigenomic analyses are available at https://github.com/NinoPandiloski.

Expanded View for this article is available online.

Acknowledgments
KAP1 KO cells and plasmids for the transcriptional silencing assay were kind gifts from Helen Rowe (University College London). The plasmids were provided with permission from David Haussler (Univ. of California Santa Cruz). pA-MNase used in CUT&RUN experiments was a kind gift from Steven Henikoff (Fred Hutchinson Cancer Research Center). We acknowledge Jenny Johansson for technical assistance with CUT&RUN library preparations. Crystallographic data were collected on beamline i04 at diamond light source (DLS). Access to DLS (proposal MX21426) was supported by the Wellcome Trust, MRC, and BBSRC. We thank Stephen McLaughlin and Chris M. Johnson for help while using the MRC-LMB Biophysics Facility. We thank Fabrice Gorrec for help in the MRC-LMB Crystallization Facility. We thank Dom Bellini for help in the MRC-LMB X-ray Crystallography Facility. This work was supported by MRC research grant MR/S021604/1 to Y.M., Wellcome Trust Senior Research Fellowship 217191/Z/19/Z to Y.M., Wellcome Trust PhD Studentship 205833/Z/16/Z to G.S., and starting grants from the Swedish Society for Medical Research (SSMF, S19-0100) and the Swedish Research Council (VR, 2021-03494) to C.H.D.

Author contributions
Guido A Stoll: Conceptualization; formal analysis; funding acquisition; investigation; visualization; methodology; writing—original draft; writing—review and editing. **Ninoslav Pandiloski:** Formal analysis; investigation; visualization. **Christopher H Douse:** Resources; data curation; formal analysis; supervision; funding acquisition; investigation; visualization; writing—review and editing. **Yorgo Modis:** Conceptualization; resources; data curation; formal analysis; supervision; funding acquisition; validation; investigation; visualization; methodology; writing—original draft; project administration; writing—review and editing.

Disclosure and competing interests statement
Y.M. is a consultant for Related Sciences LLC.

 © 2022 The Authors

References

Adams PD, Afonine PV, Bunkoczi G, Chen VB, Echols N, Headd JJ, Hung LW, Jain S, Kapral GJ, Grosse Kunstleve RW *et al* (2011) The Phenix software for automated determination of macromolecular structures. *Methods* 55: 94 – 106

Alerasool N, Segal D, Lee H, Taipale M (2020) An efficient KRAB domain for CRISPRi applications in human cells. *Nat Methods* 17: 1093 – 1096

Azebi S, Batsche E, Michel F, Kornobis E, Muchardt C (2019) Expression of endogenous retroviruses reflects increased usage of atypical enhancers in T cells. *EMBO J* 38: e101107

Bacon CW, Challa A, Hyder U, Shukla A, Borkar AN, Bayo J, Liu J, Wu SY, Chiang CM, Kutateladze TG *et al* (2020) KAP1 is a chromatin reader that couples steps of RNA polymerase II transcription to sustain oncogenic programs. *Mol Cell* 78: 1133 – 1151.e14

Cammas F, Mark M, Dolle P, Dierich A, Chambon P, Losson R (2000) Mice lacking the transcriptional corepressor TIF1beta are defective in early postimplantation development. *Development* 127: 2955 – 2963

Chuong EB, Elde NC, Feschotte C (2016) Regulatory evolution of innate immunity through co-option of endogenous retroviruses. *Science* 351: 1083 – 1087

Coluccio A, Ecco G, Duc J, Offner S, Turelli P, Trono D (2018) Individual retrotransposon integrants are differentially controlled by KZFP/KAP1-dependent histone methylation, DNA methylation and TET-mediated hydroxymethylation in naive embryonic stem cells. *Epigenetics Chromatin* 11: 7

Cowtan K, Emsley P, Wilson KS (2011) From crystal to structure with CCP4. *Acta Crystallogr D Biol Crystallogr* 67: 233 – 234

Ding D, Bergmaier P, Sachs P, Klangwart M, Ruckert T, Bartels N, Demmers J, Dekker M, Poot RA, Mermoud JE (2018) The CUE1 domain of the SNF2-like chromatin remodeler SMARCAD1 mediates its association with KRAB-associated protein 1 (KAP1) and KAP1 target genes. *J Biol Chem* 293: 2711 – 2724

Douse CH, Tchasovnikarova IA, Timms RT, Protasio AV, Seczynska M, Prigozhin DM, Albecka A, Wagstaff J, Williamson JC, Freund SMV *et al* (2020) TASOR is a pseudo-PARP that directs HUSH complex assembly and epigenetic transposon control. *Nat Commun* 11: 4940

Dupressoir A, Lavialle C, Heidmann T (2012) From ancestral infectious retroviruses to bona fide cellular genes: role of the captured syncytins in placentation. *Placenta* 33: 663 – 671

Evans R, O'Neill M, Pritzel A, Antropova N, Senior A, Green T, Žídek A, Bates R, Blackwell S, Yim J *et al* (2021) Protein complex prediction with AlphaFold-Multimer. *bioRxiv* https://doi.org/10.1101/2021.10.04.463034 [PREPRINT]

Fonti G, Marcaida MJ, Bryan LC, Trager S, Kalantzi AS, Helleboid PJ, Demurtas D, Tully MD, Grudinin S, Trono D *et al* (2019) KAP1 is an antiparallel dimer with a functional asymmetry. *Life Sci Alliance* 2: e201900349

Friedli M, Trono D (2015) The developmental control of transposable elements and the evolution of higher species. *Annu Rev Cell Dev Biol* 31: 429 – 451

Friedman JR, Fredericks WJ, Jensen DE, Speicher DW, Huang XP, Neilson EG, Rauscher FJ 3rd (1996) KAP-1, a novel corepressor for the highly conserved KRAB repression domain. *Genes Dev* 10: 2067 – 2078

Gilbert LA, Horlbeck Max A, Adamson B, Villalta Jacqueline E, Chen Y, Whitehead Evan H, Guimaraes C, Panning B, Ploegh Hidde L, Bassik Michael C *et al* (2014) Genome-scale CRISPR-mediated control of gene repression and activation. *Cell* 159: 647 – 661

Goodier JL (2016) Restricting retrotransposons: a review. *Mob DNA* 7: 16

Grow EJ, Flynn RA, Chavez SL, Bayless NL, Wossidlo M, Wesche DJ, Martin L, Ware CB, Blish CA, Chang HY *et al* (2015) Intrinsic retroviral reactivation in human preimplantation embryos and pluripotent cells. *Nature* 522: 221 – 225

Hagemans D, van Belzen IA, Moran Luengo T, Rudiger SG (2015) A script to highlight hydrophobicity and charge on protein surfaces. *Front Mol Biosci* 2: 56

Hancks DC, Kazazian HH Jr (2016) Roles for retrotransposon insertions in human disease. *Mob DNA* 7: 9

Heinz S, Benner C, Spann N, Bertolino E, Lin YC, Laslo P, Cheng JX, Murre C, Singh H, Glass CK (2010) Simple combinations of lineage-determining transcription factors prime cis-regulatory elements required for macrophage and B cell identities. *Mol Cell* 38: 576 – 589

Helleboid PY, Heusel M, Duc J, Piot C, Thorball CW, Coluccio A, Pontis J, Imbeault M, Turelli P, Aebersold R *et al* (2019) The interactome of KRAB zinc finger proteins reveals the evolutionary history of their functional diversification. *EMBO J* 38: e101220

Hung T, Pratt GA, Sundararaman B, Townsend MJ, Chaivorapol C, Bhangale T, Graham RR, Ortmann W, Criswell LA, Yeo GW *et al* (2015) The Ro60 autoantigen binds endogenous retroelements and regulates inflammatory gene expression. *Science* 350: 455 – 459

Imbeault M, Helleboid PY, Trono D (2017) KRAB zinc-finger proteins contribute to the evolution of gene regulatory networks. *Nature* 543: 550 – 554

Jacobs FM, Greenberg D, Nguyen N, Haeussler M, Ewing AD, Katzman S, Paten B, Salama SR, Haussler D (2014) An evolutionary arms race between KRAB zinc-finger genes ZNF91/93 and SVA/L1 retrotransposons. *Nature* 516: 242 – 245

Jang SM, Kauzlaric A, Quivy JP, Pontis J, Rauwel B, Coluccio A, Offner S, Duc J, Turelli P, Almouzni G *et al* (2018) KAP1 facilitates reinstatement of heterochromatin after DNA replication. *Nucleic Acids Res* 46: 8788 – 8802

Johansson PA, Brattas PL, Douse CH, Hsieh P, Adami A, Pontis J, Grassi D, Garza R, Sozzi E, Cataldo R *et al* (2022) A cis-acting structural variation at the ZNF558 locus controls a gene regulatory network in human brain development. *Cell Stem Cell* 29: 52 – 69.e8

Jumper J, Evans R, Pritzel A, Green T, Figurnov M, Ronneberger O, Tunyasuvunakool K, Bates R, Zidek A, Potapenko A *et al* (2021) Highly accurate protein structure prediction with AlphaFold. *Nature* 596: 583 – 589

Kim SS, Chen YM, O'Leary E, Witzgall R, Vidal M, Bonventre JV (1996) A novel member of the RING finger family, KRIP-1, associates with the KRAB-A transcriptional repressor domain of zinc finger proteins. *Proc Natl Acad Sci U S A* 93: 15299 – 15304

Kremer D, Gruchot J, Weyers V, Oldemeier L, Gottle P, Healy L, Ho Jang J, Kang TXY, Volsko C, Dutta R *et al* (2019) pHERV-W envelope protein fuels microglial cell-dependent damage of myelinated axons in multiple sclerosis. *Proc Natl Acad Sci U S A* 116: 15216 – 15225

Lamprecht B, Walter K, Kreher S, Kumar R, Hummel M, Lenze D, Kochert K, Bouhlel MA, Richter J, Soler E *et al* (2010) Derepression of an endogenous long terminal repeat activates the CSF1R proto-oncogene in human lymphoma. *Nat Med* 16: 571 – 579

Li W, Lee MH, Henderson L, Tyagi R, Bachani M, Steiner J, Campanac E, Hoffman DA, von Geldern G, Johnson K *et al* (2015) Human endogenous retrovirus-K contributes to motor neuron disease. *Sci Transl Med* 7: 307ra153

Li Q, Qin Y, Wang W, Jia M, Zhao W, Zhao C (2021) KAP1-mediated epigenetic suppression in anti-RNA viral responses by direct targeting RIG-I and MDA5. *J Immunol* 207: 1903 – 1910

© 2022 The Authors

Lim M, Newman JA, Williams HL, Masino L, Aitkenhead H, Gravard AE, Gileadi O, Svejstrup JQ (2019) A ubiquitin-binding domain that binds a structural fold distinct from that of ubiquitin. *Structure* 27: 1316 – 1325.e1316

Love MI, Huber W, Anders S (2014) Moderated estimation of fold change and dispersion for RNA-seq data with DESeq2. *Genome Biol* 15: 550

Margolin JF, Friedman JR, Meyer WK, Vissing H, Thiesen HJ, Rauscher FJ (1994) Kruppel-associated boxes are potent transcriptional repression domains. *Proc Natl Acad Sci U S A* 91: 4509 – 4513

McCoy AJ, Grosse-Kunstleve RW, Adams PD, Winn MD, Storoni LC, Read RJ (2007) Phasercrystallographic software. *J Appl Cryst* 40: 658 – 674

Meers MP, Tenenbaum D, Henikoff S (2019) Peak calling by sparse enrichment analysis for CUT&RUN chromatin profiling. *Epigenetics Chromatin* 12: 42

Mirdita M, Schutze K, Moriwaki Y, Heo L, Ovchinnikov S, Steinegger M (2022) ColabFold: making protein folding accessible to all. *Nat Methods* 19: 679 – 682

Mistry J, Chuguransky S, Williams L, Qureshi M, Salazar GA, Sonnhammer ELL, Tosatto SCE, Paladin L, Raj S, Richardson LJ *et al* (2021) Pfam: the protein families database in 2021. *Nucleic Acids Res* 49: D412 – D419

Moosmann P, Georgiev O, Le Douarin B, Bourquin JP, Schaffner W (1996) Transcriptional repression by RING finger protein TIF1 beta that interacts with the KRAB repressor domain of KOX1. *Nucleic Acids Res* 24: 4859 – 4867

Nicholls RA, Kovalevskiy O, Murshudov GN (2017) Low resolution refinement of atomic models against crystallographic data. *Methods Mol Biol* 1607: 565 – 593

Pastuzyn ED, Day CE, Kearns RB, Kyrke-Smith M, Taibi AV, McCormick J, Yoder N, Belnap DM, Erlendsson S, Morado DR *et al* (2018) The neuronal gene arc encodes a repurposed retrotransposon gag protein that mediates intercellular RNA transfer. *Cell* 172: 275 – 288.e218

Peng H, Gibson LC, Capili AD, Borden KLB, Osborne MJ, Harper SL, Speicher DW, Zhao K, Marmorstein R, Rock TA *et al* (2007) The structurally disordered KRAB repression domain is incorporated into a protease resistant Core upon binding to KAP-1-RBCC domain. *J Mol Biol* 370: 269 – 289

Peng H, Ivanov AV, Oh HJ, Lau YF, Rauscher FJ 3rd (2009) Epigenetic gene silencing by the SRY protein is mediated by a KRAB-O protein that recruits the KAP1 co-repressor machinery. *J Biol Chem* 284: 35670 – 35680

Robbez-Masson L, Tie CHC, Conde L, Tunbak H, Husovsky C, Tchasovnikarova IA, Timms RT, Herrero J, Lehner PJ, Rowe HM (2018) The HUSH complex cooperates with TRIM28 to repress young retrotransposons and new genes. *Genome Res* 28: 836 – 845

Rowe HM, Jakobsson J, Mesnard D, Rougemont J, Reynard S, Aktas T, Maillard PV, Layard-Liesching H, Verp S, Marquis J *et al* (2010) KAP1 controls endogenous retroviruses in embryonic stem cells. *Nature* 463: 237 – 240

Saito K, Koshiba S, Inoue M, Kigawa T, Yokoyama S, (RSGI) RSGPI (2003) Solution structure of the Kruppel-associated box (KRAB) domain

Schultz DC, Friedman JR, Rauscher FJ 3rd (2001) Targeting histone deacetylase complexes via KRAB-zinc finger proteins: THE PHD and bromodomains of KAP-1 form a cooperative unit that recruits a novel isoform of the mi-2alpha subunit of NuRD. *Genes Dev* 15: 428 – 443

Schultz DC, Ayyanathan K, Negorev D, Maul GG, Rauscher FJ 3rd (2002) SETDB1: a novel KAP-1-associated histone H3, lysine 9-specific methyltransferase that contributes to HP1-mediated silencing of euchromatic genes by KRAB zinc-finger proteins. *Genes Dev* 16: 919 – 932

Seczynska M, Bloor S, Cuesta SM, Lehner PJ (2022) Genome surveillance by HUSH-mediated silencing of intronless mobile elements. *Nature* 601: 440 – 445

Skene PJ, Henikoff JG, Henikoff S (2018) Targeted *in situ* genome-wide profiling with high efficiency for low cell numbers. *Nat Protoc* 13: 1006 – 1019

Stoll GA, Oda SI, Chong ZS, Yu M, McLaughlin SH, Modis Y (2019) Structure of KAP1 tripartite motif identifies molecular interfaces required for retroelement silencing. *Proc Natl Acad Sci U S A* 116: 15042 – 15051

Thakore PI, D'Ippolito AM, Song L, Safi A, Shivakumar NK, Kabadi AM, Reddy TE, Crawford GE, Gersbach CA (2015) Highly specific epigenome editing by CRISPR-Cas9 repressors for silencing of distal regulatory elements. *Nat Methods* 12: 1143 – 1149

Tie CH, Fernandes L, Conde L, Robbez-Masson L, Sumner RP, Peacock T, Rodriguez-Plata MT, Mickute G, Gifford R, Towers GJ *et al* (2018) KAP1 regulates endogenous retroviruses in adult human cells and contributes to innate immune control. *EMBO Rep* 19: e45000

Tycko J, DelRosso N, Hess GT, Aradhana BA, Mukund A, Van MV, Ego BK, Yao D, Spees K *et al* (2020) High-throughput discovery and characterization of human transcriptional effectors. *Cell* 183: 2020 – 2035.e2016

Varadi M, Anyango S, Deshpande M, Nair S, Natassia C, Yordanova G, Yuan D, Stroe O, Wood G, Laydon A *et al* (2022) AlphaFold protein structure database: massively expanding the structural coverage of protein-sequence space with high-accuracy models. *Nucleic Acids Res* 50: D439 – D444

Witzgall R, O'Leary E, Leaf A, Onaldi D, Bonventre JV (1994) The Kruppel-associated box-a (KRAB-A) domain of zinc finger proteins mediates transcriptional repression. *Proc Natl Acad Sci U S A* 91: 4514 – 4518

Zhou L, Mitra R, Atkinson PW, Hickman AB, Dyda F, Craig NL (2004) Transposition of hAT elements links transposable elements and V(D)J recombination. *Nature* 432: 995 – 1001

License: This is an open access article under the terms of the Creative Commons Attribution License, which permits use, distribution and reproduction in any medium, provided the original work is properly cited.

© 2022 The Authors

Expanded View Figures

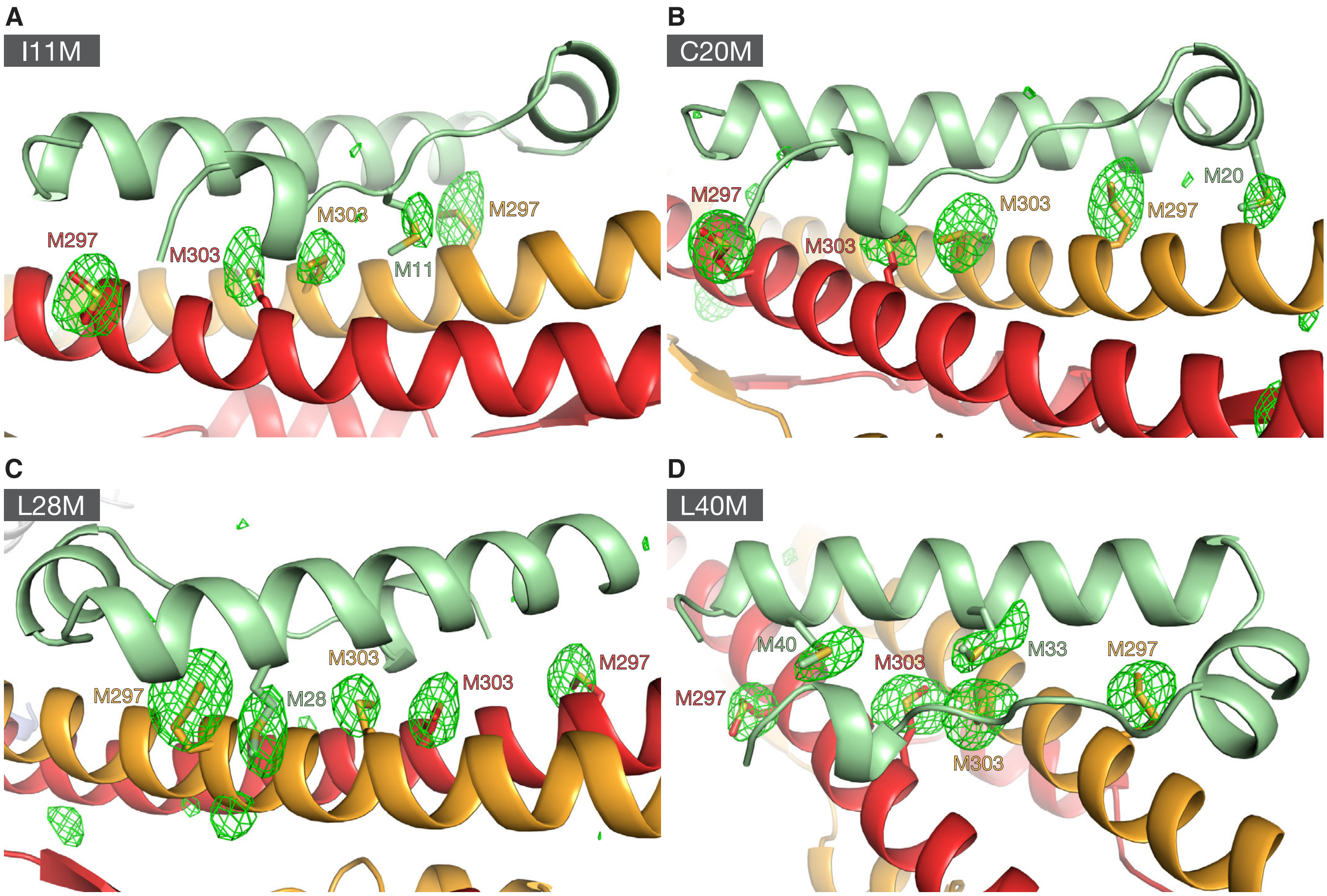

Figure EV1. Anomalous Fourier maps of selenomethionine derivatives of KAP1 RBCC in complex with four different ZNF93 methionine-insertion mutants, related to Fig 1.

A–D X-ray diffraction data were collected at the selenium K absorption edge. Anomalous Fourier maps were contoured at 3.5 σ. The Fourier maps show the positions of selenium atoms of the selenium-substituted methionine residues the KRAB and KAP1 RBCC domains. KAP1 RBCC in complex with ZNF93 KRAB I11M, (A), ZNF93 KRAB C20M, (B), ZNF93 KRAB L28M, (C), ZNF93 KRAB L40M, (D).

© 2022 The Authors

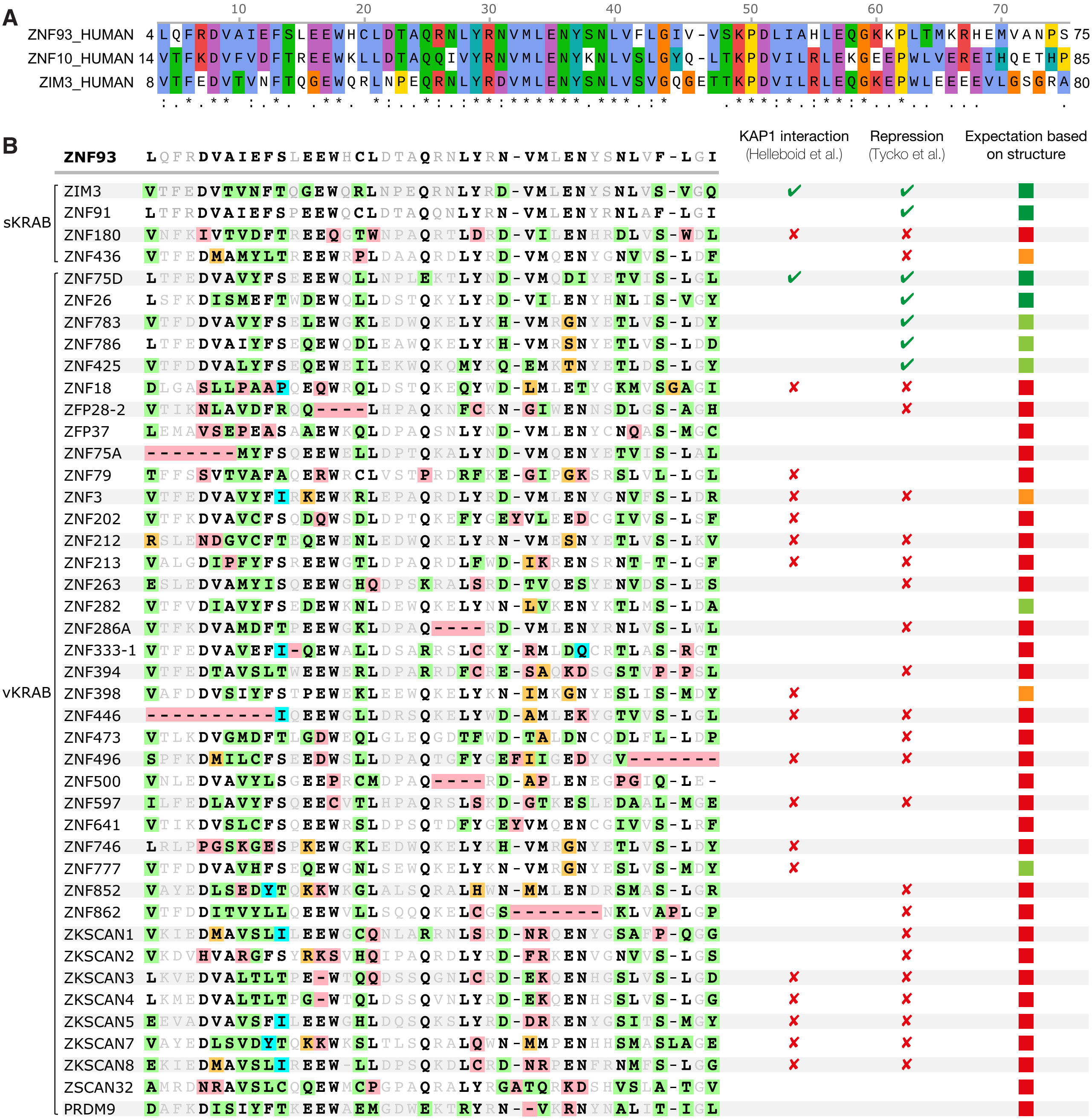

Figure EV2. Amino acid sequence alignments of KRAB domains, related to Fig 3.

A The KRAB domains from ZNF93, ZNF10, and ZIM3. Fused to catalytically inactive Cas9, the KRAB domains of ZNF10 and ZIM3 are used for potent gene repression that can be programmed in a sequence-specific manner via the CRISPRi approach (Gilbert *et al*, 2014; Thakore *et al*, 2015; Alerasool *et al*, 2020).

B Alignment of unusual KRAB domains. Variant KRAB domains (vKRAB, as classified by (Helleboid *et al*, 2019), and two standard KRAB domains (sKRAB) which were non-repressive in a high-throughput screen (Tycko *et al*, 2020), are aligned to the KRAB domain of ZNF93. ZNF93 residues directly contacting KAP1 in the crystal structure are in bold; residues not contacting KAP1 are in gray. KAP1-contacting residues that deviate from the ZNF93 sequence are highlighted in green, orange, or red, depending on whether the mutation is expected to be tolerated, moderately deleterious or highly deleterious, respectively. The right-hand column shows the expectation of whether each KRAB domain will interact with KAP1, based on our structural analysis (green, likely to bind tightly; orange, may still bind; red, unlikely to bind). The repressive sKRAB domains of ZNF91 and ZIM3 are shown for reference.

 © 2022 The Authors

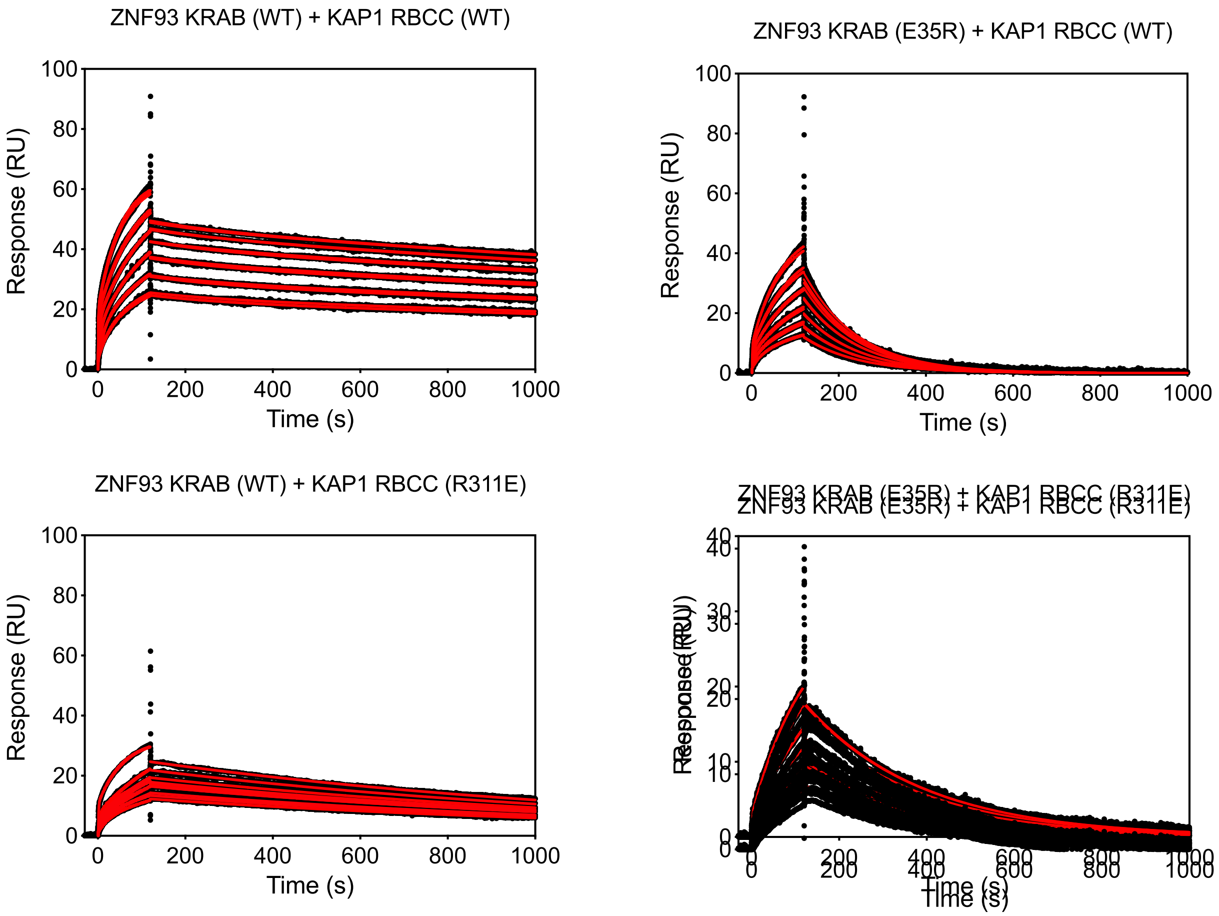

Analyte	Ligand	Phase	k_{off} (s^{-1})	k_{on} ($M^{-1} s^{-1}$)	K_d (nM)
KAP1 RBCC (WT)	ZNF93 KRAB (WT)	major	2.32×10^{-4}	3.11×10^{4}	7.5
		minor	6.1×10^{-3}	3.2×10^{2}	19×10^{3}
KAP RBCC (R311E)	ZNF93 KRAB (WT)	major	8.3×10^{-4}	2.9×10^{4}	29
		minor	-	4.2×10^{2}	2×10^{3}
KAP1 RBCC (WT)	ZNF93 KRAB (E35R)	major	8.0×10^{-3}	4.1×10^{4}	198
		minor	-	1.7×10^{2}	47×10^{3}
KAP1 RBCC (R311E)	ZNF93 KRAB (E35R)	major	3.7×10^{-3}	5.2×10^{4}	71.8
		minor	-	1.05×10^{2}	35.6×10^{3}

Figure EV3. Surface plasmon resonance (SPR) KAP1-KRAB binding assay titration curves, related to Fig 4.

Upper panel: SPR sensorgrams for KAP1 binding to immobilized MBP KRAB. The fits for the association and dissociation kinetics are shown in red. Lower panel: Data were fitted using a biphasic kinetic model with PRISM 9 (GraphPad) to determine rate constants (k_{on}, k_{off}) and binding affinities (K_d).

© 2022 The Authors

Figure EV4. Expression levels and genomic distribution of KAP1 variants in HEK239T cells, related to Fig 5.

A Heatmaps and summary plots illustrating KAP1 ChIP-seq enrichment over H3K9me3 peaks (left) and protein-coding gene promoters (right) genome wide, in KAP1 KO cells and complemented cell lines expressing WT KAP1 or KRAB binding-deficient KAP1 variant K296S/M297S/L300S/V293S (CCmut). TSS, transcriptional start site. The summary plots illustrate mean coverage values (RPKM) for each sample minus the signal of the pooled input sample. Only reads mapping uniquely (MAPQ > 10) were retained. ChIP-seq experiments were run in duplicate.

B Example genome browser snapshots of KAP1 enrichment in KAP1 KO cells complemented cell lines expressing WT or CCmut KAP1. Top, a gene with KRAB-dependent KAP1 binding at the 3′ end and KRAB-independent KAP1 binding at the promoter. Bottom, an LTR transposon bound by KAP1 in a KRAB-dependent manner. The KAP1 KO cells and a pooled input track are shown as controls. Only reads mapping uniquely (MAPQ > 10) were retained. Scales are in RPKM, reads per kilobase per million.

C Western blot of WT, KAP1 KO, and KAP1-complemented KAP1 KO HEK293T cells. These cells were used for CUT&RUN genomic profiling (Figs 5 and EV5).

D Confocal immunofluorescence microscopy of the HEK293T cells used for CUT&RUN genomic profiling stained with anti-KAP1 antibody and DAPI nuclear stain. Scale bars, 10 μm.

E Co-immunoprecipitation of KAP1 and SETDB1. KAP1 was immunoprecipitated from HEK293T KAP1 KO cells stably expressing 3xFLAG-KAP1 (WT or CCmut) using ANTI-FLAG M2 beads. The experiment was performed either in the presence or absence of the SUMO protease inhibitor N-ethylmaleimide (NEM). Uncropped blots available in Source Data.

Source data are available online for this figure.

 © 2022 The Authors

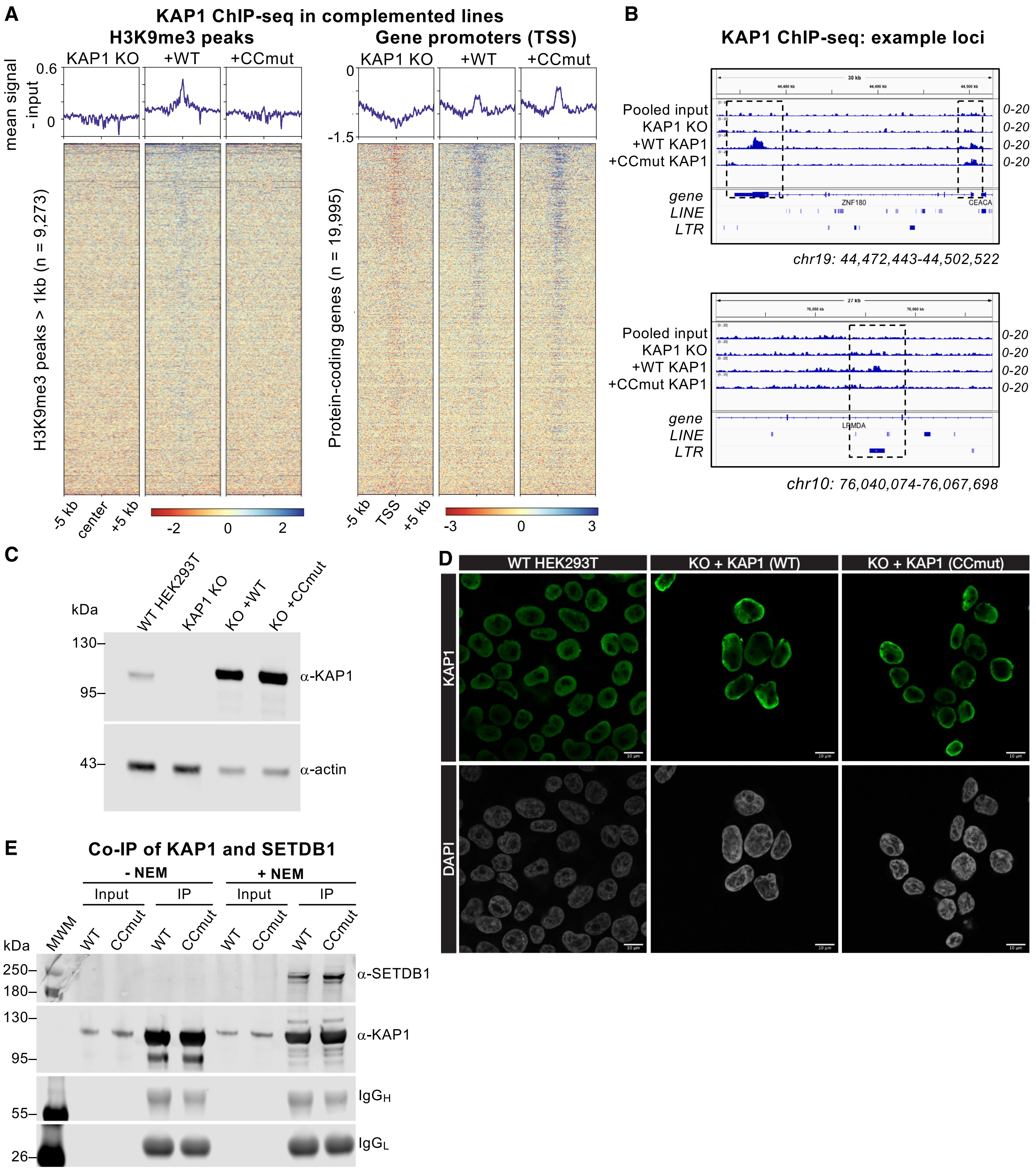
A
KAP1 ChIP-seq in complemented lines
H3K9me3 peaks
Gene promoters (TSS)
KAP1 KO
+WT
+CCmut
mean signal - input
H3K9me3 peaks > 1kb (n = 9,273)
Protein-coding genes (n = 19,995)
-5 kb
center
+5 kb
TSS
B
KAP1 ChIP-seq: example loci
Pooled input
KAP1 KO
+WT KAP1
+CCmut KAP1
gene
LINE
LTR
0-20
chr19: 44,472,443-44,502,522
chr10: 76,040,074-76,067,698
C
kDa
WT HEK293T
KAP1 KO
KO +WT
KO +CCmut
α-KAP1
α-actin
D
WT HEK293T
KO + KAP1 (WT)
KO + KAP1 (CCmut)
KAP1
DAPI
E
Co-IP of KAP1 and SETDB1
- NEM
+ NEM
Input
IP
MWM
WT
CCmut
α-SETDB1
α-KAP1
IgGH
IgGL

Figure EV4.

Figure EV5. Genome-wide analysis of H3K9me3 distribution in cells expressing wild-type and KRAB binding-deficient variants of KAP1, Related to Fig 5.

A Pairwise quantifications of H3K9me3 CUT&RUN counts for KAP1-complemented cells (n = 2) versus KAP1 KO cells (n = 2) over reference H3K9me3 peaks called in the parental control cell line. Only reads mapping uniquely (MAPQ > 10) were retained. Gray indicates significant enrichment of signal (cut-off: $P < 0.0001$; fold-change > 3) in the complemented line.

B Representative H3K9me3 distribution (over the hg38 reference) in the presence of different KAP1 variants at three different types of KAP1-independent loci: an intronic LINE-1 (L1PA) element bound by the HUSH complex (upper); a HUSH-bound long exon (middle); and a centromeric region (lower). A control IgG track from parent HEK293T cells is shown for comparison. WT, wild-type; CCmut, KRAB binding-deficient KAP1 variant. Only reads mapping uniquely (MAPQ > 10) were retained. Scales are in RPKM, reads per kilobase per million. Experiments were run in duplicate with similar results.

C Heatmaps and summary plots illustrating H3K9me3 levels over H3K9me3 peaks at HUSH-bound loci (Douse *et al*, 2020; Seczynska *et al*, 2022), in cells expressing different KAP1 variants. Peaks were called on control cells using SEACR in stringent mode (Meers *et al*, 2019). The summary plots illustrate mean values for each sample. Only reads mapping uniquely (MAPQ > 10) were retained.

D Quantitative comparison of H3K9me3 changes upon KAP1 knockout over loci regulated by HUSH (Douse *et al*, 2020; Seczynska *et al*, 2022) versus those regulated by KAP1 (as defined in this study). The central bands denote the medians. Boxes represent the interquartile range (IQR). Whiskers extend 1.5x IQR beyond the box. Statistical test: Wilcoxon rank sum test with continuity correction.

E Bar plot illustrating the distribution of loci where H3K9me3 was not reduced in KAP1 KO cells compared with the parental control cell line (KAP1-independent loci).

 © 2022 The Authors

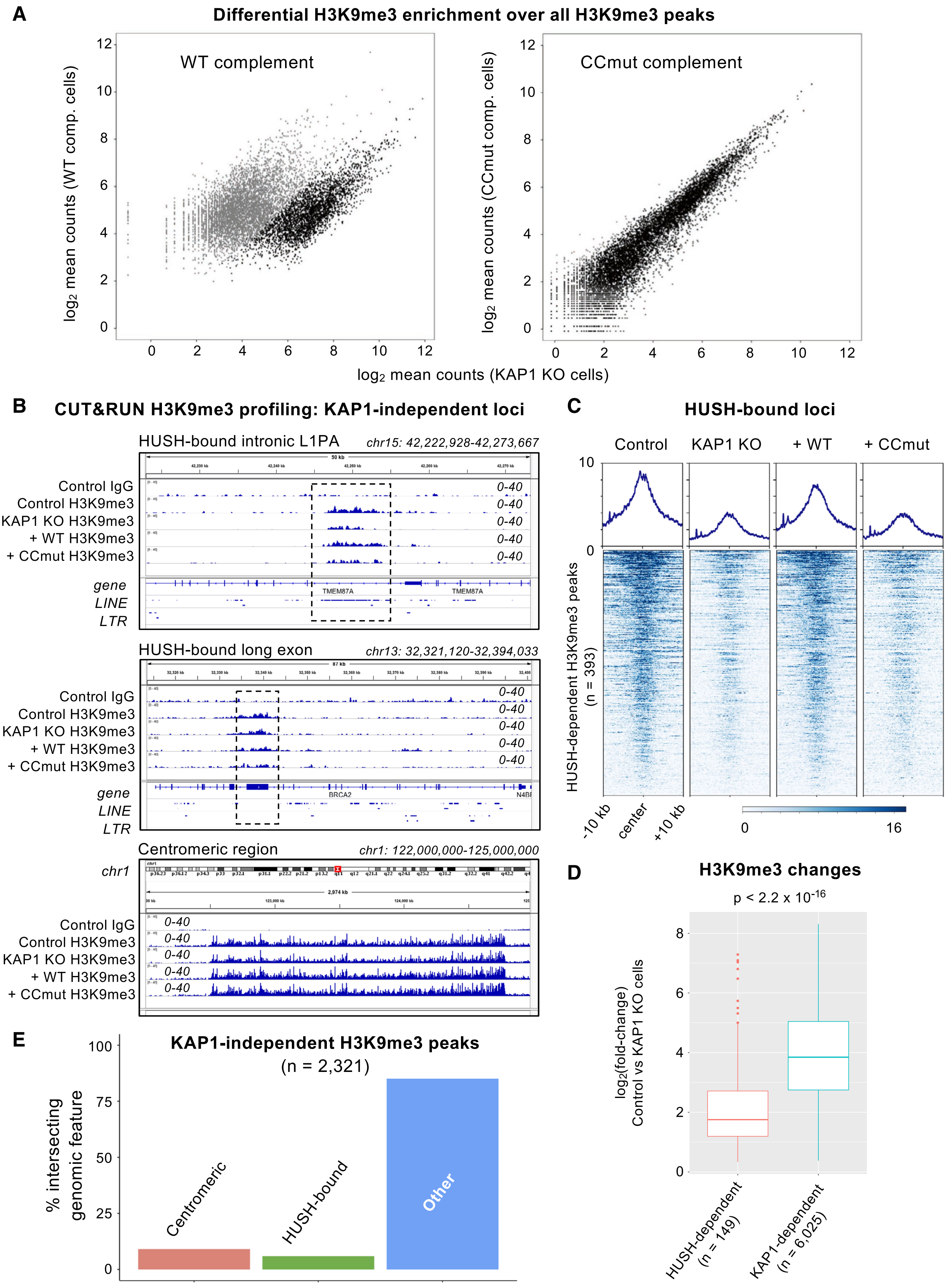

A
Differential H3K9me3 enrichment over all H3K9me3 peaks
WT complement
CCmut complement
log2 mean counts (WT comp. cells)
log2 mean counts (CCmut comp. cells)
log2 mean counts (KAP1 KO cells)
B
CUT&RUN H3K9me3 profiling: KAP1-independent loci
HUSH-bound intronic L1PA
chr15: 42,222,928-42,273,667
Control IgG
Control H3K9me3
KAP1 KO H3K9me3
+ WT H3K9me3
+ CCmut H3K9me3
gene
LINE
LTR
TMEM87A
HUSH-bound long exon
chr13: 32,321,120-32,394,033
BRCA2
Centromeric region
chr1: 122,000,000-125,000,000
chr1
0-40
C
HUSH-bound loci
Control
KAP1 KO
+ WT
+ CCmut
HUSH-dependent H3K9me3 peaks (n = 393)
-10 kb
center
+10 kb
D
H3K9me3 changes
p < 2.2 x 10-16
log2(fold-change) Control vs KAP1 KO cells
HUSH-dependent (n = 149)
KAP1-dependent (n = 6,025)
E
KAP1-independent H3K9me3 peaks
(n = 2,321)
% intersecting genomic feature
Centromeric
HUSH-bound
Other

Figure EV5.
