# Supplementary material for: Structure and functional mapping of the KRAB‐KAP1 repressor complex
Source: EMBO J. 2022 Nov 7;41(24):e111179. doi: 10.15252/embj.2022111179 (PMC9753469; doi:10.15252/embj.2022111179)
Supplement: Supplementary file 5 [file EMBJ-41-e111179-s001.pdf]

THE EMBO JOURNAL

Structure and functional mapping of the KRAB-KAP1 repressor complex

Guido Stoll, Ninoslav Pandiloski, Christopher Douse, and Yorgo Modis
DOI: 10.15252/embj.2022111179

Corresponding author(s): Yorgo Modis (ymodis@mrc-lmb.cam.ac.uk)

Review Timeline:

Submission Date:	15th Mar 22
Editorial Decision:	29th Apr 22
Revision Received:	28th Sep 22
Editorial Decision:	10th Oct 22
Revision Received:	18th Oct 22
Accepted:	20th Oct 22

Editor: Stefanie Boehm

Transaction Report:

(Note: With the exception of the correction of typographical or spelling errors that could be a source of ambiguity, letters and reports are not edited. Depending on transfer agreements, referee reports obtained elsewhere may or may not be included in this compilation. Referee reports are anonymous unless the Referee chooses to sign their reports.)

1st Editorial Decision 29th Apr 2022

Thank you for submitting your manuscript on the structural analysis of the KRAB-KAP1 interaction to The EMBO Journal. Please excuse the delay in communicating this decision to you, which was due to delayed referee responses as well as my absence from the office the last few days. We have now however received three referee reports on your study, which are included below for your information. In light of these comments, we would like to invite you to prepare and submit a revised manuscript.

As you will see, the reviewers are overall positive and appreciate the findings and their interest to the field. However, they do raise a number of points which should be addressed in the revised version of the manuscript. In particular, the KAP1-KRAB/SETDB1 complex and its recruitment should be assessed in further detail (ref#1-point 5; ref#3- major point 1), and the analysis and interpretation of the AlphaFold results discussed in more detail (ref#1- point 1; ref#2- point 2). Please also carefully consider all other referee comments and revise the manuscript and figures as needed, as well as providing a detailed response to each comment.

Referee #1:

General summary and opinion about the principal significance of the study, its questions and findings

The KRAB domain defines the largest transcription factor (TF) family in the human genome. KRAB-domain containing zinc-finger proteins (KZFPs) control the expression of transposable elements (TEs) as well as that of host genes and thereby mediate multiple key biological processes. For its versatile repressive function, the KRAB domain has been adapted to confer repression at specific genomic loci using its fusion protein with a catalytically deficient Cas9 (dCas9). This dCas9-KRAB has become a popular genetic tool known as CRISPR interference (CRISPRi) to dissect gene regulatory systems through targeting promoter or enhancer regions.

Although it has long been known that the KRAB domain deposits H3K9me3 by recruiting a scaffold protein, KAP1, and the key domains and residues important for the KRAB-KAP1 interactions have been identified, its interaction has not been solved structurally, which hampered our deeper understanding of it.

Stoll et al. (hereafter, the authors) solved the structure of the KRAB-KAP1 complex, revealing residues involved in the binding from both proteins. The authors further validated the involvement of the identified KAP1 residues in its functions by reporter assays and by H3K9me3 CUT&RUN.

This manuscript presents enough evidence to support the authors' claims. Given that the KRAB-KAP1 interaction is important for not only understanding the biology of KZFPs but also designing CRISPRi with a higher potency, we believe this work is of interest for wide research areas, although we think some minor concerns deserve to be addressed before publication.

Specific major concerns essential to be addressed to support the conclusions
We do not have any major concerns.

Minor concerns that should be addressed

1. The authors should add detailed information about the AlphaFold2 and AlphaFold-Multimer analyses so that the readers can reproduce them.

2. The Tycko et al paper is a great resource that complements this study by a high-throughput analysis of human KRAB domains and we believe the authors should take full advantage of it. By a deep mutational scan, they identified 12 KRAB A-BOX residues that significantly contribute to its silencing. Although several of them are mentioned in the manuscript for their direct involvement in the binding, the authors should summarise if any explanation could be given for the rest of the residues based on the structure they obtained

3. It is known that a phylogenetic tree of the human KRAB domains forms two major clades: one closer to the consensus (standard KRAB) and the other away from it (variant KRAB). The standard KRABs often recruit KAP1, while the variant KRABs do not; however, there are several KRABs that do not follow this trend as shown in the Tycko et al paper. Could the author explain these based on residues involved in KRAB-KAP1 binding?

4. In Fig 4C and D, the authors should clarify what comparisons were made to assess statistical significance.

5. In Fig 4C and D, it looks like some KAP1 mutants de-silence the SVA reporter more weakly than the LINE1 reporter. Is this statistically significant? If so, could this be explained by the difference in the KRAB domains of ZNF91 and ZNF93?

6. The authors show that mutations in KAP1-KRAB interacting residues abolished reporter silencing as well as genome-wide H3K9me3 deposition. This is indeed likely due to failure in KAP1-KRAB interaction, but an alternative explanation is that such mutation(s) interferes with KAP1-SETDB1 interaction. I believe that showing the mutant KAP1 still binds similar levels of SETDB1 will make the authors' claim complete.

7. In Fig 5C, the authors show that CCmut completely failed to restore the H3K9me3 levels in retrotransposons. The authors should also assess it genome-wide as it is known that some KZFPs bind other classes of TEs as well as genic regions.

8. We believe the line "Notably, H3K9me3 was more modestly reduced at sites targeted by the HUSH complex, primarily intronic LINE-1s and long exons (Douse et al, 2020; Seczynska et al, 2022) (Fig EV3)" (p.p. 14) actually refers to Fig EV4.

9. Also, the authors should show quantitative data to support further their claim, such as the difference in H3K9me3 signal between the HUSH-bound loci and the rest.

10. In EV4, the authors should show what proportion of the KAP1-independent H3K9me3 loci can be explained by HUSH-binding or centromeric regions by a bar plot.

11. In Fig 5D, the heatmap annotation for THE1 says "center" unlike the other two TEs. The authors should explain what this means. We believe that it would be more informative to show a position-aligned heatmap just like the other ones.

Additional non-essential suggestions for improving the study (which will be at the author's/editor's discretion)

12. We believe testing if ZIM3 S46F indeed strengthens its potency as speculated by the authors would be a significant improvement to this manuscript. This would not only further support the authors' claim but also contribute to rational design of a KRAB domain with higher potency, which greatly benefits its application in CRISPRi.

13. Ideally, one would like to predict if a KRAB domain binds KAP1 or not solely based on its sequence. Could the authors come up with a scoring system that roughly estimates how likely a KRAB binds KAP1 based on a given KRAB sequence?

Referee #2:

I need to state that this is not really my area, so my review is limited to my structural biology expertise and interest as a general reader. I am not familiar enough with the field to have a strong opinion on the novelty and significance.

The paper describes the structural determination of a complex between two truncated proteins (and the title should really reflect that these are not full-length proteins) that allows full biochemical characterisation of the interface between a KRAB domain (of ZNF93) and the RBCC domain of TRIM28. The key residues identified in the interface are then validated through mutagenesis and a silencing assay. The structural data are high quality, with 5 crystal structures reported, including 4 SeMet substitutions to aid sequence assignments in regions of ambiguous density. This is an important point, as in an almost entirely helical structure, the register of residues is important to get right.
I'm not totally sure I understood what the alphafold derived models add to the manuscript.
Despite not being in the area, I found the manuscript to be interesting and well-written, and the structure was not previously known, indeed the suggestion that there is likely to be significant disorder prior to complex formation is an interesting one, and one that could be worth following up experimentally.
I don't have any concerns with the structural data.

Referee #3:

This paper is of interest to readers in the fields of structural biology, chromatin and epigenetics, KRAB zinc finger proteins and transposable elements/endogenous retroviruses. The manuscript reveals the structure of the interaction of the KRAB domain of the Zinc finger protein (KZFP) ZNF93 with the RBCC domain of the common KZFP co-repressor Kap1, made possible by 1) adding the T4 lysozyme to the N terminus of the RBCC domain 2) deletion of the flexible B-box 1 domain of KAP1, and 3) adding the CUE1 domain of the chromatin remodeler SMARCAD1, which binds to the KAP1 coiled coil domain. The authors make use of recently developed Alphafold2 and Alphafold-Multimer tools to validate and compare their structural model, which make the structure convincing. In addition, they carefully assess a panel of previously established mutations that abolish

silencing both in the KRAB domain (initially identified for the related KZFP ZNF10) and in KAP1's RBCC domain (in particular V293S/K296A/M297A/L300S, individually or combined (when combined called CCmut, which the authors themselves had previously introduced and shown to abolish KRAB interactions). To demonstrate the biological importance of the interaction, the authors use KAP1 KO HEK293T cells complemented with WT and e.g. CCmut KAP1 in luciferase reporter assays to show loss of repressive activity upon mutation of the interacting residues. They also assess these complemented cells with H3K9me3 cut&run to investigate if loss of the interaction affects heterochromatin formation via the KAP1 interacting H3K9me3 histone methyltransferase SETDB1. These experiments convincingly show that KZFP interactions are indispensable for repressing transposable elements in HEK293T cells. The presented data sufficiently support the claims of the paper, are well controlled and well presented. The results present a significant advance in our understanding of KAP1 and its interactions with KZFPs, the largest transcription factor family in human. These results will allow researchers in other fields where KAP1 has essential roles (e.g. stem cell or developmental or cancer biology) to assess the importance of KZFP interactions.

Major concerns

• There have been many reports of direct interactions between components of the KRAB/KAP1/SETDB1 complexes, and this multivalency might suggest that KAP1 could be recruited independently of KRAB proteins to some chromatin domains. DO mutations that alter KRAB /KAP1 binding also prevent KAP1 binding to chromatin at ERVs/TEs? This is certainly suggested by the reduction in H3K9me3, but would be more convincing if the authors showed that KAP1/SETDB1 fails to be recruited to these sites in their "rescued cell" lines. There is an available KAP1 antibody that has been used successfully for ChIP-seq (e.g. with ab10484 used by Frank Jacobs lab)

Minor concerns:

• Page 17: KRAB-KAP1 complexes recruit multiple effectors that modify the and epigenetic conformational landscape of chromatin target loci including HP1, SETDB1, NuRD and SMARCAD1.
Remove 'and': '...modify the and epigenetic conformational landscape...'

• Page 12/Fig 4C+D: ' ...V293S [,] was not expressed at detectable levels in KAP1 KO HEK293T cells'.
If feasible it may be worth repeating the overexpression experiment, given this mutation is part of the 4 mutations used in the functional characterization (CCmut) in the remainder of the manuscript.

• I may have missed this: Is the H3K9me3 antibody used for cut&run not listed? If not: please add the antibody used.

Editor's Summary

As you will see, the reviewers are overall positive and appreciate the findings and their interest to the field. However, they do raise a number of points which should be addressed in the revised version of the manuscript. In particular, the KAP1-KRAB/ SETDB1 complex and its recruitment should be assessed in further detail (ref#1-point 5; ref#3- major point 1), and the analysis and interpretation of the AlphaFold results discussed in more detail (ref#1- point 1; ref#2- point 2). Please also carefully consider all other referee comments and revise the manuscript and figures as needed, as well as providing a detailed response to each comment.

Dear Editor, Dear Referees,

We thank all three referees for their attentive reading of this manuscript, and for their positive, constructive comments and useful suggestions.

We have carefully and extensively revised our manuscript in response to the referees' comments. Revisions include new experimental data shown in the expanded view figures, along with text edits throughout the manuscript. The most important additions and changes are the following:

- **To address the only "major concern", from Referee 3, regarding whether recruitment of KAP1 complex to chromatin is KRAB-dependent, we performed genome-wide profiling of KAP1 distribution by ChIP-seq, as suggested by Referee 3. Analysis of the ChIP-seq data showed that KAP1 enrichment in cells expressing WT KAP1 occurred at H3K9me3-positive regions and gene promoters (new figure panels Fig EV4A,B). No enrichment was observed over H3K9me3-positive regions in cells expressing a KRAB binding-deficient KAP1 variant, indicating that KAP1 recruitment to these sites depends on KRAB binding. Interestingly, WT and KRAB binding-deficient KAP1 both remained enriched over gene promoters, suggesting that KAP1 recruitment to promoters is independent of KRAB binding.**
- **We have added new immunoprecipitation data demonstrating that the KAP1-SETDB1 interaction is unaffected by mutations inhibiting KRAB binding (new figure panel Fig EV4E). This supports our hypothesis that the loss of H3K9me3 in the KRAB binding-deficient variant is due to KAP1 not being recruited to H3K9me3 loci (rather than disruption of SETDB1 binding). Moreover, we show that the KAP1-SETDB1 interaction is dependent on SUMOylation.**
- **We have added a paragraph in the Materials and Methods describing how the AlphaFold2 models were generated, specifying which installations of AlphaFold used and the URL of the server website.**
- **We have added additional new figure panels (Figs EV5A,D,E), new text in the Results (p. 7-9) and Methods (pp. 13-17), and minor edits throughout the manuscript to address the remaining minor concerns of the referees.**

We attach a copy of the manuscript with all substantive changes to the text highlighted (please note that text deleted since the previous version is not shown).

Detailed point-by-point responses to the referees' comments follow below. Thank you for your consideration.

Yorgo Modis (on behalf of the authors)

Referee #1:

General summary and opinion about the principal significance of the study, its questions and findings

The KRAB domain defines the largest transcription factor (TF) family in the human genome. KRAB-domain containing zinc-finger proteins (KZFPs) control the expression of transposable elements (TEs) as well as that of host genes and thereby mediate multiple key biological processes. For its versatile repressive function, the KRAB domain has been adapted to confer repression at specific genomic loci using its fusion protein with a catalytically deficient Cas9 (dCas9). This dCas9-KRAB has become a popular genetic tool known as CRISPR interference (CRISPRi) to dissect gene regulatory systems through targeting promoter or enhancer regions.

Although it has long been known that the KRAB domain deposits H3K9me3 by recruiting a scaffold protein, KAP1, and the key domains and residues important for the KRAB-KAP1 interactions have been identified, its interaction has not been solved structurally, which hampered our deeper understanding of it.

Stoll et al. (hereafter, the authors) solved the structure of the KRAB-KAP1 complex, revealing residues involved in the binding from both proteins. The authors further validated the involvement of the identified KAP1 residues in its functions by reporter assays and by H3K9me3 CUT&RUN.

This manuscript presents enough evidence to support the authors' claims. Given that the KRAB-KAP1 interaction is important for not only understanding the biology of KZFPs but also designing CRISPRi with a higher potency, we believe this work is of interest for wide research areas, although we think some minor concerns deserve to be addressed before publication.

Specific major concerns essential to be addressed to support the conclusions
We do not have any major concerns.

Minor concerns that should be addressed

1. The authors should add detailed information about the AlphaFold2 and AlphaFold-Multimer analyses so that the readers can reproduce them.

We have added a paragraph in the Materials and Methods describing how the AlphaFold2 models were generated including the specific installations of AlphaFold used and the URL of the server website.

2. The Tycko et al paper is a great resource that complements this study by a high-throughput analysis of human KRAB domains and we believe the authors should take full advantage of it. By a deep mutational scan, they identified 12 KRAB A-BOX residues that significantly contribute to its silencing. Although several of them are mentioned in the manuscript for their direct involvement in the binding, the authors should summarise if any explanation could be given for the rest of the residues based on the structure they obtained

Of the 12 KRAB A-Box residues identified by Tycko et al (2020), 10 were already discussed in the context of our crystal structure (specifically residues Asp8, Val9, Ile11, Phe13, Glu17, Leu21, Tyr29, Val32, Met33 and Glu35 in ZNF93 residue numbering; see pages 6 and 7). The two remaining residues are Trp18 and Asn36. Asn36 contributes to the KAP1 binding interface, forming a hydrogen bond with the side chain of Asn376. Asn36 also stabilizes the first helix of the KRAB A-box via an

intramolecular hydrogen bond. Trp18 is unusual in that although it is well conserved and was reported to be required for silencing by Tycko et al (2020), it does not contribute to the KAP1 binding interface or stabilize the KRAB domain fold based on the crystal structure. Hence our structure cannot explain why Trp18 is required for silencing. We have added a few lines in the corresponding Results section (p. 7) summarizing these observations. We have also modified Fig 3C to show Trp18 and Asn36 in the appropriate closeup views of the structure. Hence all 12 residues identified by Tycko et al are now shown in Fig 3 and discussed in the text.

3. It is known that a phylogenetic tree of the human KRAB domains forms two major clades: one closer to the consensus (standard KRAB) and the other away from it (variant KRAB). The standard KRABs often recruit KAP1, while the variant KRABs do not; however, there are several KRABs that do not follow this trend as shown in the Tycko et al paper. Could the author explain these based on residues involved in KRAB-KAP1 binding?

We thank the referee for this question. To address it, we produced a multiple sequence alignment of 39 variant KRAB domains and two unusual standard KRABs (new figure panel, Fig EV2B). We highlight residues that form contacts with KAP1. For residues that differ from the ZNF93 sequence we use a green, orange, and red color code to indicate whether they are expected to be tolerated, detrimental, or inhibitory for KAP1 binding. Based on this analysis, and consistent with the literature, most variant KRABs are not expected to bind KAP1, but a few (e.g. ZNF75D, ZNF26) are predicted to bind tightly. Our analysis extends to some KRABs for which there is no data, offering prospective predictive value.

4. In Fig 4C and D, the authors should clarify what comparisons were made to assess statistical significance.

Upon reexamining these figure panels, we opted to remove the statistical significance asterisks as the difference between WT KAP1 and other KAP1 variants is self-evident without statistical tests. All three data points are shown for each sample.

5. In Fig 4C and D, it looks like some KAP1 mutants de-silence the SVA reporter more weakly than the LINE1 reporter. Is this statistically significant? If so, could this be explained by the difference in the KRAB domains of ZNF91 and ZNF93?

The fold repression observed for WT KAP1 and the KAP1 mutants are indeed different for the LINE1 and SVA reporter. This could be due to differences in the KRAB domains of the cognate ZNFs (ZNF93 and ZFN91, respectively), as the referee suggests. Differences could include differences in KAP1 binding affinity, DNA binding affinity, ZNF expression level, or the epigenetic background of the reporter. Because the relative (and absolute) repression observed with WT KAP1 is different in the two assays, direct statistical comparisons of the KAP1 mutants across the two assays (e.g. with a t-test or Anova test) would not be appropriate. In this context, although the KAP1 M297A mutant may be less derepressed than the other mutants in the SVA reporter, we refrain from speculating on the basis of this difference.

6. The authors show that mutations in KAP1-KRAB interacting residues abolished reporter silencing as well as genome-wide H3K9me3 deposition. This is indeed likely due to failure in KAP1-KRAB interaction, but an alternative explanation is that such mutation(s) interferes with KAP1-SETDB1 interaction. I believe that showing the mutant KAP1 still binds similar levels of SETDB1 will make the authors' claim complete.

The referee raises a valid point. We have addressed this by performing co-immunoprecipitation experiments of KAP1 and SETDB1 from cell lysates. These

pulldowns showed that WT KAP1 and a KRAB binding-deficient variant (CCmut) recruit SETDB1 equally well (see new figure panel Fig EV4E), demonstrating that SETDB1 recruitment by KAP1 is independent of KRAB binding. Moreover, the SETDB1-KAP1 interaction was only observed in the presence of the SUMO protease inhibitor *N*-ethylmaleimide (NEM), which confirms that KAP1 recruits SETDB1 in a SUMO-dependent manner.

7. In Fig 5C, the authors show that CCmut completely failed to restore the H3K9me3 levels in retrotransposons. The authors should also assess it genome-wide as it is known that some KZFPs bind other classes of TEs as well as genic regions.

We show in Fig 5B heatmaps that show H3K9me3 levels in peaks genome-wide in the four conditions (Control, KO, WT-complemented and CCmut-complemented). We have added a new figure panel, Fig EV5A, to quantify this observation. Quantification of H3K9me3 enrichment shows that around 60% of H3K9me3 peaks genome-wide are restored in the WT-complement but not the CCmut-complement. This corresponds to essentially all those H3K9me3 peaks lost in the KO compared to the parental (control) line.

8. We believe the line "Notably, H3K9me3 was more modestly reduced at sites targeted by the HUSH complex, primarily intronic LINE-1s and long exons (Douse et al, 2020; Seczynska et al, 2022) (Fig EV3)" (p.p. 14) actually refers to Fig EV4.

Thank you for spotting this typo- it has been corrected (note that Fig EV4 from the original submission is now Fig EV5).

9. Also, the authors should show quantitative data to support further their claim, such as the difference in H3K9me3 signal between the HUSH-bound loci and the rest.

We assessed changes in H3K9me3 in control and KAP1 KO cells over two sets of H3K9me3 rich loci: KAP1-dependent loci and those bound by HUSH (based on Douse et al, 2020 and Seczynska et al., 2022). A new figure panel (Fig EV5D) shows that there is significantly smaller average reduction in H3K9me3 levels over the HUSH-bound loci. That there is measurable reduction in H3K9me3 levels over HUSH-bound loci in the KAP1 KO may reflect the cooperation between the two complexes at certain loci, as detailed in Robbez-Masson *et al* (2018; cited in the manuscript). We have added a sentence to the text to mention this conclusion (p. 9).

10. In EV4, the authors should show what proportion of the KAP1-independent H3K9me3 loci can be explained by HUSH-binding or centromeric regions by a bar plot.

A bar plot has been added as requested in a new figure panel, Fig EV5E.

11. In Fig 5D, the heatmap annotation for THE1 says "center" unlike the other two TEs. The authors should explain what this means. We believe that it would be more informative to show a position-aligned heatmap just like the other ones.

Fig 5D has been amended as requested to show a position-aligned (+/- 5kb) heatmap for all three TE types (based on the THE1 consensus sequence).

Additional non-essential suggestions for improving the study (which will be at the author's/editor's discretion)

12. We believe testing if ZIM3 S46F indeed strengthens its potency as speculated by the authors would be a significant improvement to this manuscript. This would not only further

support the authors' claim but also contribute to rational design of a KRAB domain with higher potency, which greatly benefits its application in CRISPRi.

We agree it will be useful to test if ZIM3 S46F indeed strengthens its transcriptional repression potency for CRISPRi applications. We plan to do this in future studies.

13. Ideally, one would like to predict if a KRAB domain binds KAP1 or not solely based on its sequence. Could the authors come up with a scoring system that roughly estimates how likely a KRAB binds KAP1 based on a given KRAB sequence?

A scoring system to rank and predict KAP1 binding by KRAB domains would indeed by useful. To do this effectively we believe we would need to increase the size of our dataset and examine additional KRAB domains as even with 40 or so KRABs it remains unclear what the rules should be for a scoring algorithm. We hope to incorporate this in a future study.

Referee #2:

I need to state that this is not really my area, so my review is limited to my structural biology expertise and interest as a general reader. I am not familiar enough with the field to have a strong opinion on the novelty and significance.

The paper describes the structural determination of a complex between two truncated proteins (and the title should really reflect that these are not full-length proteins) that allows full biochemical characterisation of the interface between a KRAB domain (of ZNF93) and the RBCC domain of TRIM28. The key residues identified in the interface are then validated through mutagenesis and a silencing assay. The structural data are high quality, with 5 crystal structures reported, including 4 SeMet substitutions to aid sequence assignments in regions of ambiguous density. This is an important point, as in an almost entirely helical structure, the register of residues is important to get right.

We thank the Referee for their positive overall review.

I'm not totally sure I understood what the alphafold derived models add to the manuscript. Despite not being in the area, I found the manuscript to be interesting and well-written, and the structure was not previously known, indeed the suggestion that there is likely to be significant disorder prior to complex formation is an interesting one, and one that could be worth following up experimentally.

We have added a paragraph in the Materials and Methods describing how the AlphaFold2 models were generated including the specific installations of AlphaFold used and the URL of the server website (as also requested by Referee 1).

I don't have any concerns with the structural data.

Referee #3:

This paper is of interest to readers in the fields of structural biology, chromatin and epigenetics, KRAB zinc finger proteins and transposable elements/endogenous retroviruses. The manuscript reveals the structure of the interaction of the KRAB domain of the Zinc finger protein (KZFP) ZNF93 with the RBCC domain of the common KZFP co-repressor Kap1, made possible by 1) adding the T4 lysozyme to the N terminus of the RBCC domain 2) deletion of the flexible B-box 1 domain of KAP1, and 3) adding the CUE1 domain of the chromatin remodeler SMARCAD1, which binds to the KAP1 coiled coil domain. The authors make use of recently developed Alphafold2 and Alphafold-Multimer tools to validate and compare their structural model, which make the structure convincing. In addition, they carefully assess a panel of previously established mutations that abolish silencing both in the KRAB domain (initially identified for the related KZFP ZNF10) and in KAP1's RBCC domain (in particular V293S/K296A/M297A/L300S, individually or combined (when combined called CCmut, which the authors themselves had previously introduced and shown to abolish KRAB interactions). To demonstrate the biological importance of the interaction, the authors use KAP1 KO HEK293T cells complemented with WT and e.g. CCmut KAP1 in luciferase reporter assays to show loss of repressive activity upon mutation of the interacting residues. They also assess these complemented cells with H3K9me3 cut&run to investigate if loss of the interaction affects heterochromatin formation via the KAP1 interacting H3K9me3 histone methyltransferase SETDB1. These experiments convincingly show that KZFP interactions are indispensable for repressing transposable elements in HEK293T cells. The presented data sufficiently support the claims of the paper, are well controlled and well presented. The results present a significant advance in our understanding of KAP1 and its interactions with KZFPs, the largest transcription factor family in human. These results will allow researchers in other fields where KAP1 has essential roles (e.g. stem cell or developmental or cancer biology) to assess the importance of KZFP interactions.

Major concerns

• There have been many reports of direct interactions between components of the KRAB/KAP1/SETDB1 complexes, and this multivalency might suggest that KAP1 could be recruited independently of KRAB proteins to some chromatin domains. DO mutations that alter KRAB /KAP1 binding also prevent KAP1 binding to chromatin at ERVs/TEs? This is certainly suggested by the reduction in H3K9me3, but would be more convincing if the authors showed that KAP1/SETDB1 fails to be recruited to these sites in their "rescued cell" lines. There is an available KAP1 antibody that has been used successfully for ChIP-seq (e.g. with ab10484 used by Frank Jacobs lab)

We agree with the reviewer and wanted from the beginning to probe KAP1-chromatin interactions in addition to using the indirect H3K9me3 readout. Indeed, to directly test our model that the CCmut KAP1 fails to be recruited to chromatin via the KRAB domain, we made extensive attempts to get KAP1 CUT&RUN and CUT&Tag to work at an early stage of this project (and since). All these attempts have so far have been unsuccessful, perhaps due to KAP1-chromatin interactions being indirect.

We therefore reverted to crosslinking approaches and assessed KAP1 chromatin association using KAP1 ChIP-seq, as suggested by the Referee, in KAP1-KO (negative control), WT-complemented and CCmut-complemented lines (new figure panels Fig EV4A,B).

The ChIP-seq experiment showed relatively weak KAP1 enrichment over background compared to the cleaner H3K9me3 readout. Because of this we restricted ourselves to a relatively simple analysis.

We found that KAP1 enrichment in the WT-complemented cells occurred at two types of loci: H3K9me3-positive regions and gene promoters (Fig EV4A,B). No KAP1 enrichment was observed over H3K9me3-positive regions in the CCmut-complemented cells, indicating that KAP1 recruitment to these sites depends on KRAB binding. By contrast, KAP1 remained enriched over gene promoters in the CCmut-complemented cells, suggesting that KAP1 recruitment to promoters does not depend on KRAB binding (Fig EV4A). We are unsure of the significance of the promoter peaks, but we note that KAP1-promoter interactions involving the PHD-bromodomain region of KAP1 have been reported previously (e.g. Bacon et al, 2020, now cited in the manuscript). We cannot, however, discount the possibility that the promoter peaks are an artifact of overexpression and crosslinking of the recombinant protein to open chromatin (relative expression levels in WT cells and KAP1-complemented KAP1 KO cells are shown in Fig EV4C,D). It is outside the scope of this manuscript to follow up on this, but we report the finding in the Results section with appropriate caveats (p. 8). Nonetheless, the ChIP-seq data provide some additional support for the model that KRAB binding is necessary for KAP1 recruitment at H3K9me3-positive regions. Moreover, we now show with new immunoprecipitation experiments that the KAP1-SETDB1 interaction is unaffected by CCmut (see new figure panel Fig EV4E), which further supports our hypothesis that the loss of H3K9me3 in the CCmut variant is due to KAP1 not being recruited to those loci.

Minor concerns:

• Page 17: KRAB-KAP1 complexes recruit multiple effectors that modify the and epigenetic conformational landscape of chromatin target loci including HP1, SETDB1, NuRD and SMARCAD1.
 Remove 'and': '...modify the and epigenetic conformational landscape...'

Typo corrected, thank you.

• Page 12/Fig 4C+D: ' ...V293S [,] was not expressed at detectable levels in KAP1 KO HEK293T cells'.
 If feasible it may be worth repeating the overexpression experiment, given this mutation is part of the 4 mutations used in the functional characterization (CCmut) in the remainder of the manuscript.

We thank the reviewer for this comment. In attempting to repeat the overexpression experiment for the V293S mutant we discovered a frameshift mutation upon re-sequencing the original plasmid. This spontaneous frameshift mutation was probably responsible for the lack of expression of the original V293S construct in HEK293 cells. Due to time and staffing limitations, we were not able to complete a new set of luciferase reporter assays with the V293S single mutant, but this data would not have changed any of the conclusions in this paper. We have deleted the phrase stating that V293S single mutant was not expressed (p. 8).

• I may have missed this: Is the H3K9me3 antibody used for cut&run not listed? If not: please add the antibody used.

We thank the referee for spotting this omission. We used rabbit anti-H3K9me3, abcam cat. no. ab8898, RRID:AB_306848 or guinea pig anti-rabbit IgG (American Research Products, cat. no. CSB-PA00150E1Gp) at 1:100 dilution. This information is now listed in the Methods section for CUT&RUN.

Thank you for submitting your revised manuscript. We have now received comments from two of the initial there referees (please see below) and I am pleased to say that they now support publication. Therefore, I would like to ask you to please resolve a number of editorial issues that are listed in detail below. If you have any further questions regarding the revision or if any of the specific points are unclear, please contact us. Once these final issues are resolved, we will be happy to formally accept the study.

Referee #1:

All my concerns have been addressed.

Referee #3:

The authors addressed my concerns. I recommend publication.

2nd Author Response 10th Oct 2022

All editorial and formatting issues were resolved by the authors.

2nd Revision - Editorial Decision 20th Oct 2022

Thank you again for submitting the final revised version of your manuscript and addressing the remaining points. I am pleased to inform you that we have now accepted it for publication in The EMBO Journal.

EMBO Press Author Checklist

Corresponding Author Name: Yorgo Modis
Journal Submitted to: The EMBO Journal
Manuscript Number: EMBOJ-2022-111179

USEFUL LINKS FOR COMPLETING THIS FORM
The EMBO Journal - Author Guidelines
EMBO Reports - Author Guidelines
Molecular Systems Biology - Author Guidelines
EMBO Molecular Medicine - Author Guidelines

Reporting Checklist for Life Science Articles (updated January
This checklist is adapted from Materials Design Analysis Reporting (MDAR) Checklist for Authors. MDAR establishes a minimum set of requirements in transparent reporting in the life sciences (see Statement of Task: 10.31222/osf.io/9sm4x). Please follow the journal's guidelines in preparing your
Please note that a copy of this checklist will be published alongside your article.

Abridged guidelines for figures
1. Data
The data shown in figures should satisfy the following conditions:
- the data were obtained and processed according to the field’s best practice and are presented to reflect the results of the experiments in an accurate and unbiased manner.
- ideally, figure panels should include only measurements that are directly comparable to each other and obtained with the same assay.
- plots include clearly labeled error bars for independent experiments and sample sizes. Unless justified, error bars should not be shown for technical
- if n<5, the individual data points from each experiment should be plotted. Any statistical test employed should be justified.
- Source Data should be included to report the data underlying figures according to the guidelines set out in the authorship guidelines on Data

2. Captions
Each figure caption should contain the following information, for each panel where they are relevant:
- a specification of the experimental system investigated (eg cell line, species name).
- the assay(s) and method(s) used to carry out the reported observations and measurements.
- an explicit mention of the biological and chemical entity(ies) that are being measured.
- an explicit mention of the biological and chemical entity(ies) that are altered/varied/perturbed in a controlled manner.
- the exact sample size (n) for each experimental group/condition, given as a number, not a range;
- a description of the sample collection allowing the reader to understand whether the samples represent technical or biological replicates (including how many animals, litters, cultures, etc.).
- a statement of how many times the experiment shown was independently replicated in the laboratory.
- definitions of statistical methods and measures:
  - common tests, such as t-test (please specify whether paired vs. unpaired), simple χ2 tests, Wilcoxon and Mann-Whitney tests, can be unambiguously identified by name only, but more complex techniques should be described in the methods section;
  - are tests one-sided or two-sided?
  - are there adjustments for multiple comparisons?
  - exact statistical test results, e.g., P values = x but not P values < x;
  - definition of ‘center values’ as median or average;
  - definition of error bars as s.d. or s.e.m.

Please complete ALL of the questions below.
Select "Not Applicable" only when the requested information is not relevant for your study.

Materials

Newly Created Materials	Information included in the manuscript?	In which section is the information available? (Reagents and Tools Table, Materials and Methods, Figures, Data Availability Section)
New materials and reagents need to be available; do any restrictions apply?	Yes	Materials and Methods

Antibodies	Information included in the manuscript?	In which section is the information available? (Reagents and Tools Table, Materials and Methods, Figures, Data Availability Section)
For **antibodies** provide the following information:<br>- Commercial antibodies: RRID (if possible) or supplier name, catalogue number and or/clone number<br>- Non-commercial: RRID or citation	Yes	Materials and Methods

DNA and RNA sequences	Information included in the manuscript?	In which section is the information available? (Reagents and Tools Table, Materials and Methods, Figures, Data Availability Section)
**Short novel DNA or RNA including primers, probes**: provide the sequences.	Not Applicable	

Cell materials	Information included in the manuscript?	In which section is the information available? (Reagents and Tools Table, Materials and Methods, Figures, Data Availability Section)
**Cell lines:** Provide species information, strain. Provide accession number in repository **OR** supplier name, catalog number, clone number, and/**OR** RRID.	Yes	Materials and Methods
**Primary cultures:** Provide species, strain, sex of origin, genetic modification status.	Not Applicable	
Report if the cell lines were recently **authenticated** (e.g., by STR profiling) and tested for mycoplasma contamination.	Yes	Materials and Methods

Experimental animals	Information included in the manuscript?	In which section is the information available? (Reagents and Tools Table, Materials and Methods, Figures, Data Availability Section)
**Laboratory animals or Model organisms:** Provide species, strain, sex, age, genetic modification status. Provide accession number in repository **OR** supplier name, catalog number, clone number, **OR** RRID.	Not Applicable	
**Animal observed in or captured from the field:** Provide species, sex, and age where possible**.**	Not Applicable	
Please detail **housing and husbandry conditions**.	Not Applicable	

Plants and microbes	Information included in the manuscript?	In which section is the information available? (Reagents and Tools Table, Materials and Methods, Figures, Data Availability Section)
**Plants:** provide species and strain, ecotype and cultivar where relevant, unique accession number if available, and source (including location for collected wild specimens).	Not Applicable	
**Microbes:** provide species and strain, unique accession number if available, and source.	Yes	Materials and Methods

Human research participants	Information included in the manuscript?	In which section is the information available? (Reagents and Tools Table, Materials and Methods, Figures, Data Availability Section)
If collected and within the bounds of privacy constraints report on age, sex and gender or ethnicity for all study participants.	Not Applicable	

Core facilities	Information included in the manuscript?	In which section is the information available? (Reagents and Tools Table, Materials and Methods, Figures, Data Availability Section)
If your work benefited from core facilities, was their service mentioned in the acknowledgments section?	Yes	Acknowledgements

Design

Study protocol	Information included in the manuscript?	In which section is the information available? (Reagents and Tools Table, Materials and Methods, Figures, Data Availability Section)
If study protocol has been **pre-registered, provide DOI in the manuscript**. For clinical trials, provide the trial registration number **OR** cite DOI.	Not Applicable	
Report the **clinical trial registration number** (at ClinicalTrials.gov or equivalent), where applicable.	Not Applicable	

Laboratory protocol	Information included in the manuscript?	In which section is the information available? (Reagents and Tools Table, Materials and Methods, Figures, Data Availability Section)
Provide DOI OR other citation details if **external detailed step-by-step protocols** are available.	Not Applicable	

Experimental study design and statistics	Information included in the manuscript?	In which section is the information available? (Reagents and Tools Table, Materials and Methods, Figures, Data Availability Section)
Include a statement about **sample size** estimate even if no statistical methods were used.	Yes	Materials and Methods
Were any steps taken to minimize the effects of subjective bias when allocating animals/samples to treatment (e.g. **randomization procedure**)? If yes, have they been described?	Not Applicable	
Include a statement about **blinding** even if no blinding was done.	Yes	
Describe **inclusion/exclusion criteria** if samples or animals were excluded from the analysis. Were the criteria pre-established? If sample or data points were omitted from analysis, report if this was due to attrition or intentional exclusion and provide justification.	Not Applicable	
For every figure, are **statistical tests** justified as appropriate? Do the data meet the assumptions of the tests (e.g., normal distribution)? Describe any methods used to assess it. Is there an estimate of variation within each group of data? Is the variance similar between the groups that are being statistically compared?	Yes	Materials and Methods, Figure Legends

Sample definition and in-laboratory replication	Information included in the manuscript?	In which section is the information available? (Reagents and Tools Table, Materials and Methods, Figures, Data Availability Section)
In the figure legends: state number of times the experiment was **replicated** in laboratory.	Yes	Figure Legends
In the figure legends: define whether data describe **technical or biological replicates**.	Yes	Figure Legends

Ethics

Ethics	Information included in the manuscript?	In which section is the information available? (Reagents and Tools Table, Materials and Methods, Figures, Data Availability Section)
Studies involving **human participants**: State details of **authority granting ethics approval** (IRB or equivalent committee(s), provide reference number for approval.	Not Applicable	
Studies involving **human participants**: Include a statement confirming that **informed consent** was obtained from all subjects and that the experiments conformed to the principles set out in the WMA Declaration of Helsinki and the Department of Health and Human Services Belmont Report.	Not Applicable	
Studies involving **human participants:** For publication of **patient photos**, include a statement confirming that consent to publish was obtained.	Not Applicable	
Studies involving experimental **animals**: State details of **authority granting ethics approva**l (IRB or equivalent committee(s), provide reference number for approval. Include a statement of compliance with ethical regulations.	Not Applicable	
Studies involving **specimen and field samples:** State if relevant **permits** obtained, provide details of authority approving study; if none were required, explain why.	Not Applicable	

Dual Use Research of Concern (DURC)	Information included in the manuscript?	In which section is the information available? (Reagents and Tools Table, Materials and Methods, Figures, Data Availability Section)
Could your study fall under dual use research restrictions? Please check biosecurity documents and list of **select agents and toxins** (CDC): https://www.selectagents.gov/sat/list.htm	Not Applicable	
If you used a select agent, is the security level of the lab appropriate and reported in the manuscript?	Not Applicable	
If a study is subject to dual use research of concern regulations, is the name of the **authority granting approval and reference number** for the regulatory approval provided in the manuscript?	Not Applicable	

Reporting

The MDAR framework recommends adoption of discipline-specific guidelines, established and endorsed through community initiatives. Journals have their own policy about requiring specific guidelines and recommendations to complement MDAR.

Adherence to community standards	Information included in the manuscript?	In which section is the information available? (Reagents and Tools Table, Materials and Methods, Figures, Data Availability Section)
State if relevant guidelines or checklists (e.g., **ICMJE, MIBBI, ARRIVE, PRISMA**) have been followed or provided.	Not Applicable	
For **tumor marker prognostic studies,** we recommend that you follow the **REMARK** reporting guidelines (see link list at top right). See author guidelines, under 'Reporting Guidelines'. Please confirm you have followed these guidelines.	Not Applicable	
For **phase II and III randomized controlled trials**, please refer to the **CONSORT** flow diagram (see link list at top right) and submit the CONSORT checklist (see link list at top right) with your submission. See author guidelines, under 'Reporting Guidelines'. Please confirm you have submitted this list.	Not Applicable	

Data Availability

Data availability	Information included in the manuscript?	In which section is the information available? (Reagents and Tools Table, Materials and Methods, Figures, Data Availability Section)
Have **primary datasets** been deposited according to the journal's guidelines (see 'Data Deposition' section) and the respective accession numbers provided in the Data Availability Section?	Yes	Data Availability
Were **human clinical and genomic datasets** deposited in a public access-controlled repository in accordance to ethical obligations to the patients and to the applicable consent agreement?	Not Applicable	
Are **computational models** that are central and integral to a study available without restrictions in a machine-readable form? Were the relevant accession numbers or links provided?	Yes	Methods and Data Availability
If publicly available data were reused, provide the respective **data citations in the reference list**.	Not Applicable	
